# *NLG1*, encoding a mitochondrial membrane protein, controls leaf and grain development in rice

**DOI:** 10.1186/s12870-023-04417-2

**Published:** 2023-09-09

**Authors:** Yi Wen, Kaixiong Wu, Bingze Chai, Yunxia Fang, Peng Hu, Yiqing Tan, Yueying Wang, Hao Wu, Junge Wang, Li Zhu, Guangheng Zhang, Zhenyu Gao, Deyong Ren, Dali Zeng, Lan Shen, Guojun Dong, Qiang Zhang, Qing Li, Qian Qian, Jiang Hu

**Affiliations:** 1 Rice Research Institute of Shenyang Agricultural University/Key Laboratory of Northern Japonica Rice Genetics and Breeding, Ministry of Education and Liaoning Province, Shenyang, 110866 China; 2 State Key Laboratory of Rice Biology and Breeding, China National Rice Research Institute, Hangzhou, 310006 China; 3 College of Life and Environmental Sciences, Hangzhou Normal University, 16 Xiasha Road, Hangzhou, 310036 China; 4 Hainan Yazhou Bay Seed Laboratory, Sanya, Hainan 572024 China; 5 National Nanfan Research Institute (Sanya), Chinese Academy of Agricultural Sciences, Sanya, 572024 China

**Keywords:** NLG1, Mitochondria, Leaf width, Grain size, Auxin, Rice

## Abstract

**Background:**

Mitochondrion is the key respiratory organ and participate in multiple anabolism and catabolism pathways in eukaryote. However, the underlying mechanism of how mitochondrial membrane proteins regulate leaf and grain development remains to be further elucidated.

**Results:**

Here, a mitochondria-defective mutant *narrow leaf and slender grain 1* (*nlg1*) was identified from an EMS-treated mutant population, which exhibits narrow leaves and slender grains. Moreover, *nlg1* also presents abnormal mitochondria structure and was sensitive to the inhibitors of mitochondrial electron transport chain. Map-based cloning and transgenic functional confirmation revealed that *NLG1* encodes a mitochondrial import inner membrane translocase containing a subunit Tim21. GUS staining assay and RT-qPCR suggested that NLG1 was mainly expressed in leaves and panicles. The expression level of respiratory function and auxin response related genes were significantly down-regulated in *nlg1*, which may be responsible for the declination of ATP production and auxin content.

**Conclusions:**

These results suggested that NLG1 plays an important role in the regulation of leaf and grain size development by maintaining mitochondrial homeostasis. Our finding provides a novel insight into the effects of mitochondria development on leaf and grain morphogenesis in rice.

**Supplementary Information:**

The online version contains supplementary material available at 10.1186/s12870-023-04417-2.

## Background

Leaf morphology is a critical component of ideal plant architecture, providing a main site for photosynthesis, respiration and transpiration in rice. Appropriate leaf width is indispensable for improving photosynthetic efficiency and crop yield [1]. Up to now, dozens of genes related to leaf width development have been reported, involving in the transcription factors, auxin synthesis and signal transduction, cellulose synthesis, etc. [2]. Among them, *AUXIN RESPONSE FACTORS* (*ARFs*) and *WUSCHEL-RELATED HOMEOBOX* (*WOX*) genes are two main transcription factors controlling leaf width. The overexpression of *OsARF19* and its downstream gene *OsGH3-5* lead to a decrease in free auxin content, resulting in narrow leaf phenotype [3]. Loss of function of *OsARF11* produces narrow leaves, along with a decrease in the ratio of leaf width to leaf length, which is a typical auxin deficiency or insensitive phenotype [4]. *Narrow Leaf1* (*NAL1*) positively regulates auxin polar transport via *OsPIN1*, affecting vascular bundle arrangement and leaf width [5, 6]. *NAL2* and *NAL3* encode OsWOX3A transcription activators, and its double mutant *nal2*/*nal3* reveals extremely narrow leaves [7, 8]. *NAL7* is a member of YUCCA gene family and involved in tryptophan-dependent IAA biosynthesis, regulating leaf width and rolling [9, 10]. *NAL21* encodes ribosomal small subunit protein RPS3A and plays a role in regulating the transcription of auxin response factors *ARFs* and *OsWOX3A* to maintain the normal leaf morphology [11]. Moreover, *NAL8* encodes a prohibitin complex 2α subunit and is essential for morphogenesis of chloroplasts and mitochondria, affecting leaf width and spikelet number development [12]. *NAL22* encodes a Maf-like nucleoside triphosphate pyrophosphatase protein and its knock-out mutant displays a short and narrow leaf phenotype [13]. In addition, cellulose synthase-like protein D4 gene *Narrow and Rolled Leaf 1* (*NRL1*) is necessary for cell-wall formation and plays a vital role in leaf morphogenesis [14, 15]. Mitochondrial structural protein DECREASED VASCULAR BUNDLE1 (DVB1) participates in the organization of cristae in mitochondria. The loss-of-function mutant *dvb1* also shows narrow leaves [16]. *Abnormal Vascular Bundles* (*AVB*) encodes a novel protein with unknown biochemical function, which interacts with RL14 and regulates leaf blade development [17, 18]. QTL *qFLT9* associated with leaf thickness were fine-mapped in the 928-kb region on chromosome 9 using the F_2_ population derived from the cross between high leaf thickness variety AXZ and thin leaf thickness variety YD6, providing a novel insight into leaf morphology [19].

Grain size is a key yield trait that determined by several factors [20]. Narrow leaf mutants usually produce reduced cell size, which is closely related to the reduction of grain width and length. The SHAQKYF-class MYB transcription factor SLL1/AH2 plays a role in the determination of grain size and leaf morphology, its null mutant exhibits smaller grains and highly incurved narrow leaves [21, 22]. *GL7* is a major QTL controlling grain length. The over-expression of *GL7* leads to decreased transverse cell extension and increased longitudinal cell elongation, resulting in slender grains and leaves [23]. Narrow leaf gene *NAL2*/*OsWOX3A* acts in the development of various organs, such as leaf, spikelet, tiller and lateral root. The double mutation of *NAL2* and *NAL3* leads to pleiotropic effects, including narrow-thin grain and narrow-curly leaves [7]. *NRL1* plays a critical role in leaf morphogenesis throughout regulating cell-wall formation. Disruption of *NRL1* presents growth retardation, including declined leaf and grain width [14, 24]. *NRL2* encodes an unknown biochemical function protein that regulates fundamental cell differentiation. The loss-of-function mutant *nrl2* shows narrow leaves and slender grains [17].

The main site of cellular respiration is occurred in mitochondria, where is responsible for the homeostasis between energy production and metabolic process in all eukaryotic cells [25]. As the central source of ATP, mitochondria participate in various anabolic and catabolic processes. Its dysfunction leads to a variety of abnormal physiological and biochemical functions in rice [26]. *FLOURY ENDOSPERM10* (*FLO10*) and *FLOURY ENDOSPERM18* (*FLO18*) encodes a mitochondrial-localized P-type PPR protein, which plays a key role in endosperm development and mitochondrial function. Loss-of-functions of *FLO10* and *FLO18* leads to a floury endosperm, along with abnormal mitochondria morphology and decreased ATP content [27, 28]. Mitochondrion-targeted single-stranded DNA-binding protein TA1/OsmtSSB1 inhibits the illegitimate recombination of mtDNA in aleurone cell layers and maintains the efficient energy supply of mitochondria by interacting with mitochondrial DNA recombinase RECA3 and DNA helicase TWINKLE. The *ta1* mutant shows altered mitochondrial structure and compromised ATP content in aleurone [29]. *WHITE PANICLE3* (*WP3*) encodes a novel nucleus-encoded mitochondrial protein whose functionally disruption leads to defect in mitochondria and chloroplast development, resulting in white-striped leaf and white panicle [30].

Most of mitochondrial precursor proteins are synthesized in the cytosol and then transfer into mitochondria with the cooperation of mitochondrial membrane system [31, 32]. The Mitochondrial membranes are composed of outer and inner membrane layers. Driven by the presequence translocase-associated motor (PAM), the preproteins are imported by outer membrane (TOM) complex and insert into inner membrane or matrix with the mediation of inner membrane (TIM23) complex [33]. TIM23 complex consists of three essential subunits, Tim21, Tim23 and Tim50, which functions as a central junction in preprotein translocation. It is known that Tim21 directly binds to Tom22 subunit of TOM complex and release preproteins, thereby maintaining the connection between TIM23 and TOM complex [34, 35]. In *Arabidopsis*, there are three Tim21 proteins, SD3 (Segregation Distortion 3, AT4G00026), At2g40800 and At3g56430. Among them, SD3 is a homolog of Tim21. The *sd3* mutant seedling shows seedling-lethal under light, short hypocotyls under dark and decreased intracellular ATP level in dark-grown [36]. Here, we identified and characterized a rice mutant *nlg1*, which exhibits narrow leaf and slender grain phenotype. *NLG1* encodes a mitochondrial inner membrane translocase Tim21 that is required for the translocation of preproteins from cytosol to mitochondria. Further analysis reveals an aberrant mitochondria ultrastructure and reduced ATP contents in *nlg1* leaves and spikelet hulls, suggesting that NLG1 is involved in the maintenance of mitochondria morphology and respiratory chain. Our results revealed that *NLG1* plays an important role in leaf and grain development by maintaining mitochondria metabolism.

## Materials and methods

### Plant materials, growth conditions and phenotype characterization

The *nlg1* mutant was isolated from an ethyl methane sulfonate (EMS)-treated population of YD32 (YunDao32), which is a conventional *japonica* variety in China. All rice plant materials were cultivated under standard growth conditions in the paddy of Hangzhou (Zhejiang Province) and Lingshui (Hainan Province), China. The plant height, tiller number, internodes length, grains per panicle, grain width, grain length, leaf width and leaf length of YD32 and *nlg1* were measured at maturity stage. The ImageJ software [37] were used for measuring tissue parameters, including thickness of culm, cell layer number in a culm, spikelet hull perimeter, cell width and cell length of inner glume.

### Paraffin sectioning

Paraffin sections were conducted as previously described [38]. Briefly, the fixed rice tissues were dehydrated in a graded ethanol series, infiltrated with xylene series, embedded in paraffin and sliced in 8 ~ 10 μm thick sections, dewaxed in xylene, stained with 1% safranin and 1% Fast Green, and finally observed by using Nikon ECLIPSE 90i microscope.

### Transmission electron microscopy (TEM) and scanning electron microscopy (SEM)

For TEM, the flag leaves of *nlg1* and YD32 were collected and fixed in 2.5% glutaraldehyde fixative solution for 2 days. The fixed samples were washed with phosphate buffer saline 3 times and then post-fixed in 1% OsO_4_ solution for 1 h, followed with a uranyl acetate staining, gradient ethanol dehydration, embedded in Spurr and sliced with Leica EM UC7 ultratome. Finally, the 70 nm sectioning samples were stained again and observed under a Hitachi H-7500 Transmission Electron Microscope. For SEM, the fresh samples were observed with a Hitachi SU3500 Scanning Electron Microscope.

### Map-based Cloning of *NLG1*

The mapping population was derived from a cross between *nlg1* mutant and Taichung Native 1 (TN1) (*Oryza sativa* L. subsp. *indica*). The individuals showed mutant phenotypes in F_2_ population were selected and new InDel markers were designed from Rice Genomic Research Program database (rice.uga.edu) for *NLG1* mapping. The gene was finally fine-mapped to a 42.1-kb interval on chromosome 3, and total of 6 candidate genes were amplified and sequenced. The primers used for gene mapping are listed in Table S2.

### Vector construction and transformation

For genetic complementation, the entire sequence of *NLG1* driven by its native promoter were amplified from YD32 and fused into the *pCAMBIA1300* binary vector. For RNA interference (RNAi), a 300-bp coding region and its reverse direction were inserted into the *SacI*/*SpeI* and *KpnI*/*BamHI* restriction sites of the *pTCK303* vector respectively according to previously described method [39]. For expression pattern verification, a 2.5-kb promoter region upstream of ATG were amplified and cloned into *pCAMBIA1305.1* vector to generate *proNLG1::GUS* construction. The full coding sequence of *NLG1* without the stop codon was introduced into the *pUbi::GFP* to create p*Ubi::NLG1::GFP* overexpression vector. All above plasmids were transformed into rice callus to generate corresponding transgenic plants via agrobacterium-mediated transformation. The primers used are listed in Table S2.

### RNA extraction and RT-qPCR

Total RNA was isolated from various tissues and then complementary DNA (cDNA) syntheses were performed according to previously described method [40]. The cDNA was used as template for reverse transcription quantitative PCR (RT-qPCR) analysis by SYBR Green Real-time PCR Master Mix (Toyobo, Japan) in Applied Biosystems 7900HT Fast Real-Time PCR System. The relative expression levels were evaluated through cycle threshold (Ct) method with the 2^−ΔΔCt^ values using *OsActin (LOC_Os03g50885)* as internal reference [41]. Three biological triplicates were conducted in experiments and significant differences were analyzed via Student’s *t*-test. All primers used are given in Table S2.

### RNA-seq analysis

Total RNA from YD32 and *nlg1* were extracted using TRIzol Reagent following the manufacturer’s instructions (Invitrogen). The library preparation, RNA sequencing and data analysis were performed according to previous reports [42, 43] with some modifications. Differentially expressed genes (DEGs) were identified via edgeR with FDR < 0.05 and |log2(Fold change)| > 1 using normalized expression values.

### GUS staining assay and subcellular localization

For GUS staining assay, various tissues from transgenic plants expressing GUS driven by the promoter of *NLG1* were collected and immersed in GUS staining buffer at 37℃ for 12–16 h. The stained tissues were decolorized with 50% ethanol and then photographed under stereo microscope. To determine the subcellular localization of NLG1, the p*Ubi::NLG1::GFP* vector were co-transformed with CellLight™ Mitochondria-RFP (Invitrogen, USA) into rice protoplasts and incubated at 28℃ overnight following the instrument described [44]. GFP and RFP fluorescence were observed under LSM 700 confocal microscope (ZEISS).

### Phylogenetic analysis

The amino acid sequence of NLG1 and its 22 homologs in other species were obtained from NCBI (https://www.ncbi.nlm.nih.gov/) and presented in Supplementary File 1. Phylogenetic tree was constructed using a MEGA-X [45] with a Neighbor-joining method and each statistically significant difference value besides the branches was calculated from 1000 bootstrap replications.

### ATP and IAA content measurement

The ATP extraction and content measurement was performed with ATP Assay Kit (Beyotime, China). The 0.2-g fresh leaf and panicle tissues of YD32 and *nlg1* were homogenized with 1 ~ 2 ml lysate, and centrifuged at 12,000 g under 4 °C for 5 min. The supernatants were used to detect ATP content using GloMax® 20/20 Luminometer System (Promega, USA). Fresh leaf primordium in seedling stage were collected for IAA content measurement, and the estimations were conducted according to the reported method [16].

## Results

### Identification of *nlg1* mutant

**Fig. 1:**
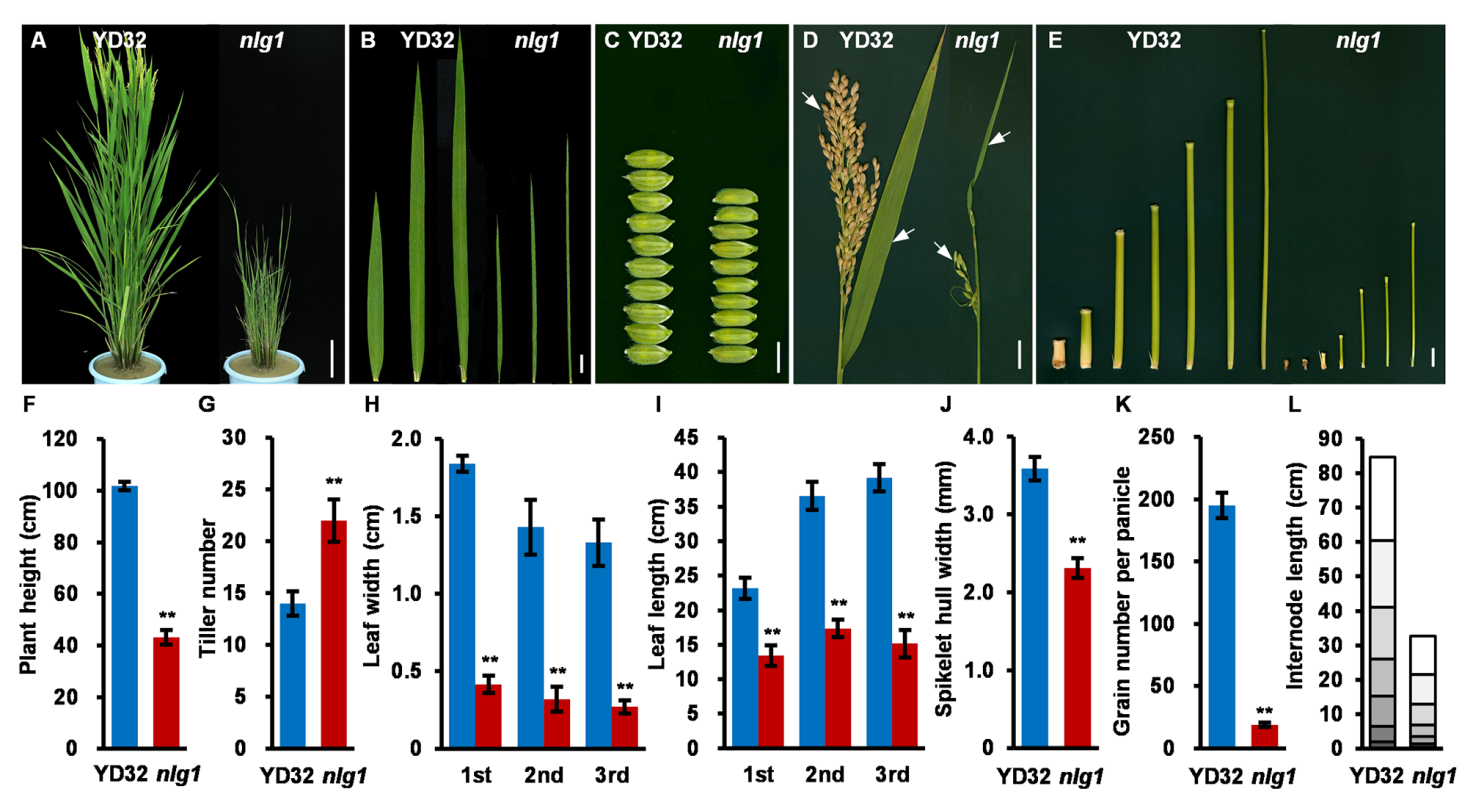
Phenotypic characterization of the wild-type YD32 and *nlg1* mutant. **A** Plant architecture of the YD32 (left) and *nlg1* (right) at heading stage. **B** 1st - 3rd leaf blades of the YD32 and *nlg1* during heading stage. **C** Spikelet hulls of the YD32 and *nlg1*. D Panicles of the YD32 and *nlg1.***E** 1st - 7th internodes of the YD32 and *nlg1.***F-L** Comparison between YD32 and *nlg1* in plant height (**F**), tiller number (**G**), leaf width (**H**), leaf length (**I**), spikelet hull width (**J**), grain number per panicle (**K**), internode length (**L**). Data represent means ± SD (*n* = 10). **Significant difference at p < 0.01 compared with YD32 by Student’s *t*-test. Scale bars: 10 cm in **A**; 2 cm in **B**, 5 mM in **C**, 2 cm in **D-E**

The *nlg1* was isolated from a mutagenesis population of *Japonica* rice cultivar YD32 induced by EMS. Under natural field environment, *nlg1* exhibited pleiotropic phenotypes, including narrow leaves, slender grains, increased tiller number and plant dwarfism (Fig. 1). Compared with the wild-type YD32, the flag leaf, second leaf and third leaf width in *nlg1* were reduced by approximately 77.4%, 77.6% and 79.7%, and the leaf length were reduced by about 42.1%, 52.5% and 61.4%, respectively (Fig. 1B, H, I). Moreover, the spikelet hull of *nlg1* was significantly narrower than that of YD32, with a decrease of 35.6% (Fig. 1C, J), and grain number per panicle was also declined by 90.3% in *nlg1* (Fig. 1D, K). The reduction of plant height in *nlg1* was mainly caused by the decrease of first to seventh internode length, which decreased by 53.9%, 55.2%, 59.3%, 70.2%, 74.9%, 78% and 78.6%, respectively (Fig. 1E, L). We also investigated the dynamic characteristics of YD32 and *nlg1* seedlings from 10 days to 35 days, and found that the leaf width and plant height difference began to appear around 10 days and 20 days after sowing (Fig. S1), respectively. However, the difference of tiller number emerged around 30 days (Fig. S1C). In general, the mutation of *NLG1* gene affects multiple growth and development process, especially in leaf width, grain size and plant height.

### *NLG1* affects the development of vascular bundles and spikelet cell size

**Fig. 2:**
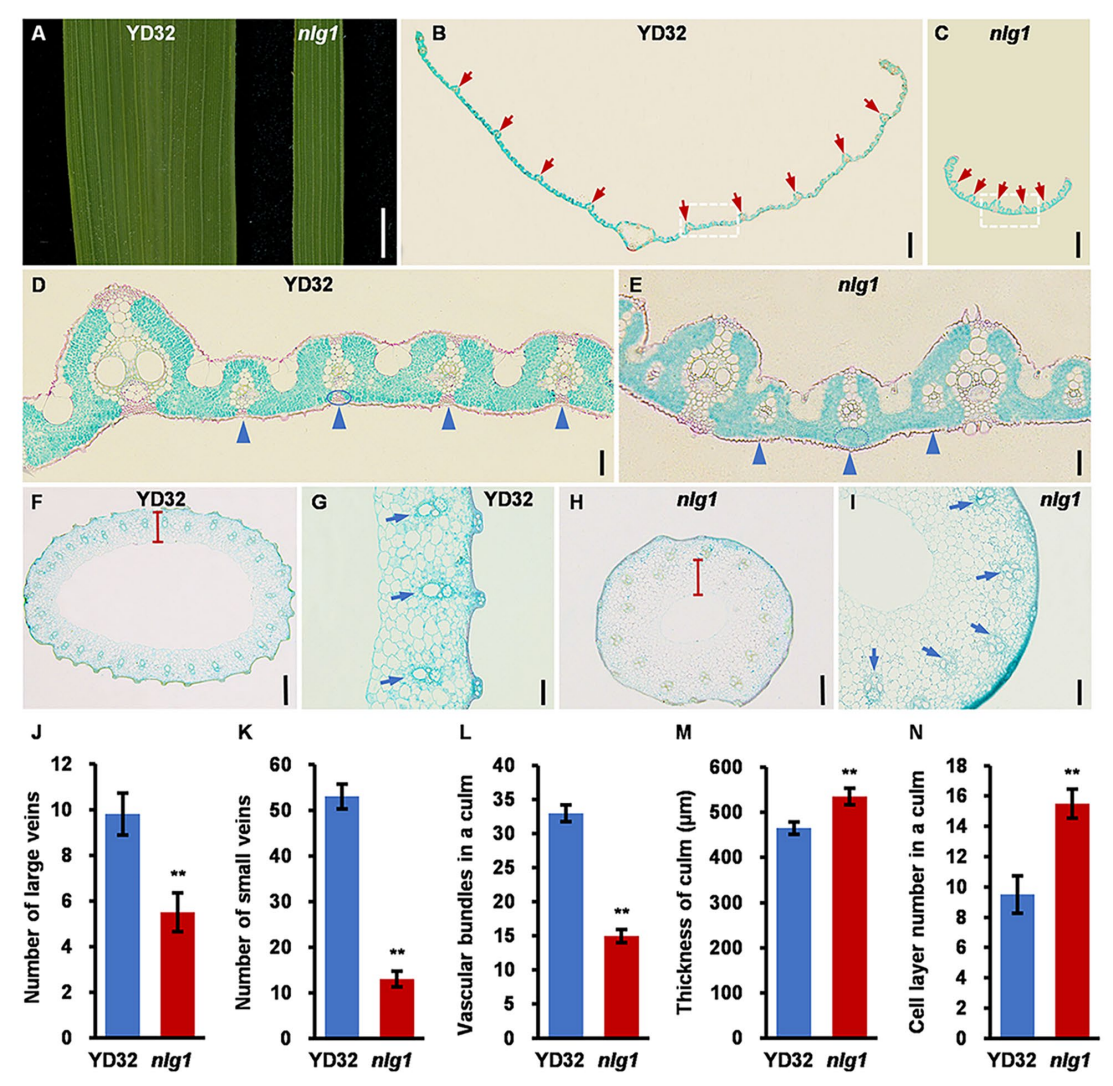
*NLG1* affects the development of vascular bundles. **A** Flag leaves of YD32 and *nlg1*. **B-C** Paraffin transverse sections of YD32 (**B**) and *nlg1* (**C**) flag leaves. Red arrows indicate large vascular bundles (LVs). **D-E** Magnified views of transverse sections of YD32 (**D**) and *nlg1* (**E**) flag leaves from white dashed boxes in **B** and **C**. blue triangles indicate small vascular bundles (SVs), blue circles indicate abaxial sclerenchyma. **F-I** Paraffin transverse sections of YD32 (**F**) and *nlg1* (**H**) second internodes. Magnified views of transverse sections of YD32 (**G**) and *nlg1* (**I**) second internodes. Blue arrows indicate vascular bundles in culm. **J-N** Comparison between YD32 and *nlg1* in number of large veins (**J**), number of small veins (**K**), vascular bundles in a culm (**L**), thickness of culm (**M**), cell layer number in a culm (**N**). Data represent means ± SD (*n* = 10). **Significant difference at p < 0.01 compared with YD32 by Student’s *t*-test. Scale bars: 5 mm in **A**; 600 μm in **B-C**; 50 μm in **D-E**; 400 μm in **F** and **H**; 100 μm in **G** and **I**

**Fig. 3:**
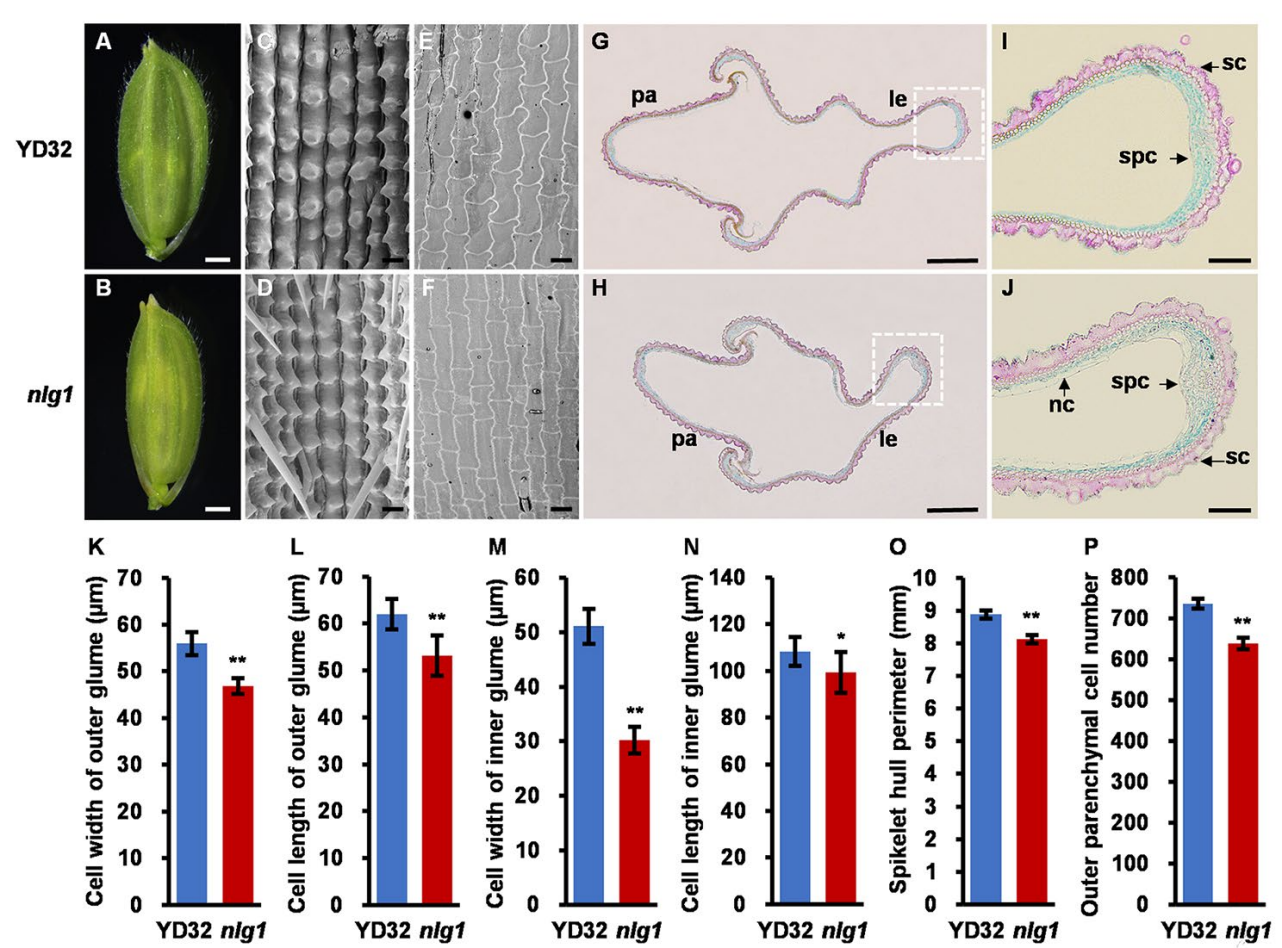
*NLG1* affects the development of spikelet cell size. **A-B** Spikelet hulls before filling of YD32 (**A**) and *nlg1* (**B**). **C-F** Scanning electron micrographs of outer and inner glumes from YD32 (**C-E**) and *nlg1* (**D-F**). **G-H** Paraffin transverse sections of YD32 (**G**) and *nlg1* (**H**) spikelet hull. le, lemma. p.a., palea. **I-J** Magnified views of transverse sections from YD32 (**I**) and *nlg1* (**J**) spikelet hull from white dashed boxes in **G** and **H**. sc, silicified cells. nc, non-silicified cells. spc, spongy parenchymatous cells. **K-P** Comparison between YD32 and *nlg1* in cell width of outer glume (**K**), cell length of outer glume (**L**), cell width of inner glume (**M**), cell length of inner glume (**N**), spikelet hull perimeter (**O**), outer parenchymal cell number (**P**), Data represent means ± SD (*n* = 10). **Significant difference at p < 0.01 and *difference at p < 0.05 compared with YD32 by Student’s *t*-test. Scale bars: 1 mm in **A-B**; 50 μm in **C-F**; 400 μm in **G-H**; 100 μm in **I-J**

To reveal the potential cytological characteristics, the vascular bundle characteristics and cell morphology in leaf, culm and spikelet were analyzed by paraffin section and SEM. The cross section of leaves showed that the number of large veins and small veins were significantly decreased by 43.9% and 75.5%, respectively, and the abaxial sclerenchyma of small veins were disappeared in *nlg1* (Fig. 2A-E, J-K). The cross section of culms revealed that the number of vascular bundles decreased by 54.5%, and the thickness of culm and number of cell layers were increased by 15.1% and 63.2% in *nlg1*, respectively (Fig. 2F-I, L-N). We also noticed that the spikelet hull width of *nlg1* was slender than YD32 (Fig. 3A-B). Consistent with the phenotype, the SEM observation showed that the average cell width and length of epidermal cells in the outer and inner glumes were significantly declined in *nlg1* (Fig. 3C-F, K-N). The paraffin section further showed that the spikelet hull perimeter and the number of outer parenchymal cells were also significantly decreased in *nlg1* (Fig. 3G-J, O-P). Taken together, these results demonstrated that the narrow leaves of *nlg1* were caused by the reduction of large veins and small veins, while the slender grains of *nlg1* were due to the decrease of cell proliferation and cell expansion.

### Map-Based cloning *NLG1*

**Fig. 4:**
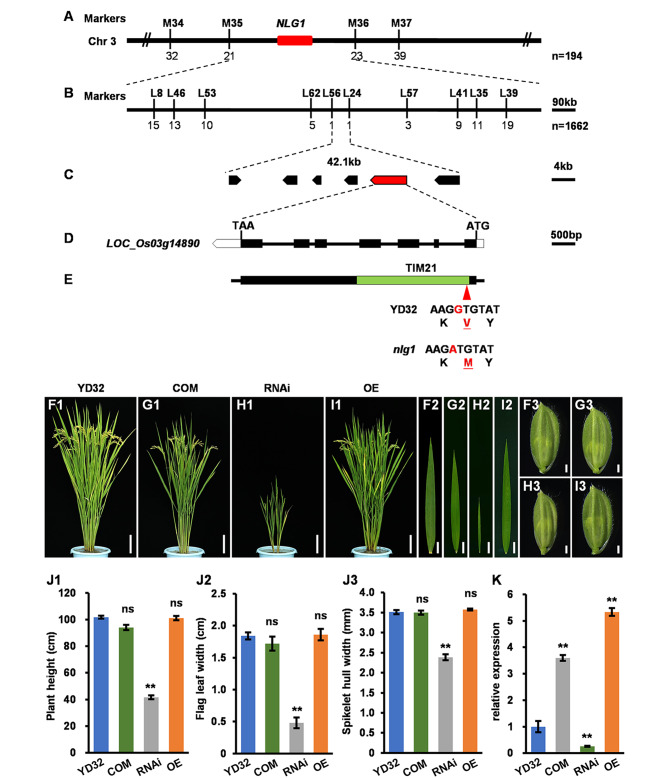
Map-based cloning and functional confirmation of *NLG1*. **A-C** Mapping of *NLG1* in a 42.1-kb region on chromosome 3. The numbers below the markers represents the recombinants. **D-E** A single base-pair substitution from G to A occurred in seventh exon of *LOC_Os03g14890*, leading to a residue alteration from Val to Met in TIM21 domain. **F-I** Morphological comparison of plant architectures, flag leaves and spikelet hulls of YD32 (F1, F2 and F3), COM (*NLG1*-complementation) (G1, G2 and G3), RNAi (*NLG1*-RNA interference) (H1, H2 and H3), OE (*NLG1*-overexpression) (I1, I2 and I3) transgenic lines. **J1-J3** Plant height (J1), flag leaf width (J2) and spikelet hull width (J3) of YD32, COM, RNAi and OE transgenic lines. Data represent means ± SD (*n* = 5). **K** Expression analysis of *NLG1* in the flag leaves of the YD32, COM, RNAi and OE transgenic lines using RT-qPCR. Data represent means ± SD (*n* = 3). **Significant difference at p < 0.01 compared with YD32 by Student’s *t*-test, and ns means no significance. Scale bars: 10 cm in F1-I1, 2 cm in F2-I2, 1 mm in F3-I3

To isolate the target gene *NLG1*, we employed a map-based cloning approach using 1856 F_2_ individuals derived from the cross between *nlg1* and TN1. *NLG1* was primitively located on chromosome 3 and finally narrowed in an interval of 42.1-kb, which contained 6 open reading frames (ORFs) based on the Rice Genome Annotation Project database (http://rice.plantbiology.msu.edu/) (Fig. 4A-C, Table S1). The 6 ORFs were sequenced and a G to A single base-pair substitution were found in the seventh exon of *LOC_Os03g14890*, causing the 227th residue alteration from valine (Val) to methionine (Met) (Fig. 4D-E). To verify the candidate gene, the entire coding sequence of *LOC_Os03g14890* harboring 2302-bp upstream of ATG and 412-bp downstream were constructed into *pCAMBIA1300* and transformed into *nlg1*. A total of 14 independent transgenic lines were obtained, and their mutant traits were all restored as YD32 (Fig. 4F, G, J; Fig. S2A, C, E; Fig. S3). In addition, we performed an RNAi suppression of *NLG1* in YD32 and found that the positive lines also presented a narrow leaves and slender grains phenotype similar to *nlg1* (Fig. 4H, J; Fig. S2B, E; Fig. S3). However, the phenotypes of *NLG1* overexpression lines were not significantly different from that of wild-type YD32 (Fig. 4I, J; Fig. S2D, E; Fig. S3). We further detected the transcription level of *NLG1* in these lines and found that *NLG1* were up-regulated in overexpression and complementary lines and were down-regulated markedly in RNAi lines (Fig. 4K; Fig. S2F, G). These results above indicated that *LOC_Os03g14890* was identical to *NLG1*.

### *NLG1* encodes a mitochondrial import inner membrane translocase Tim21

**Fig. 5:**
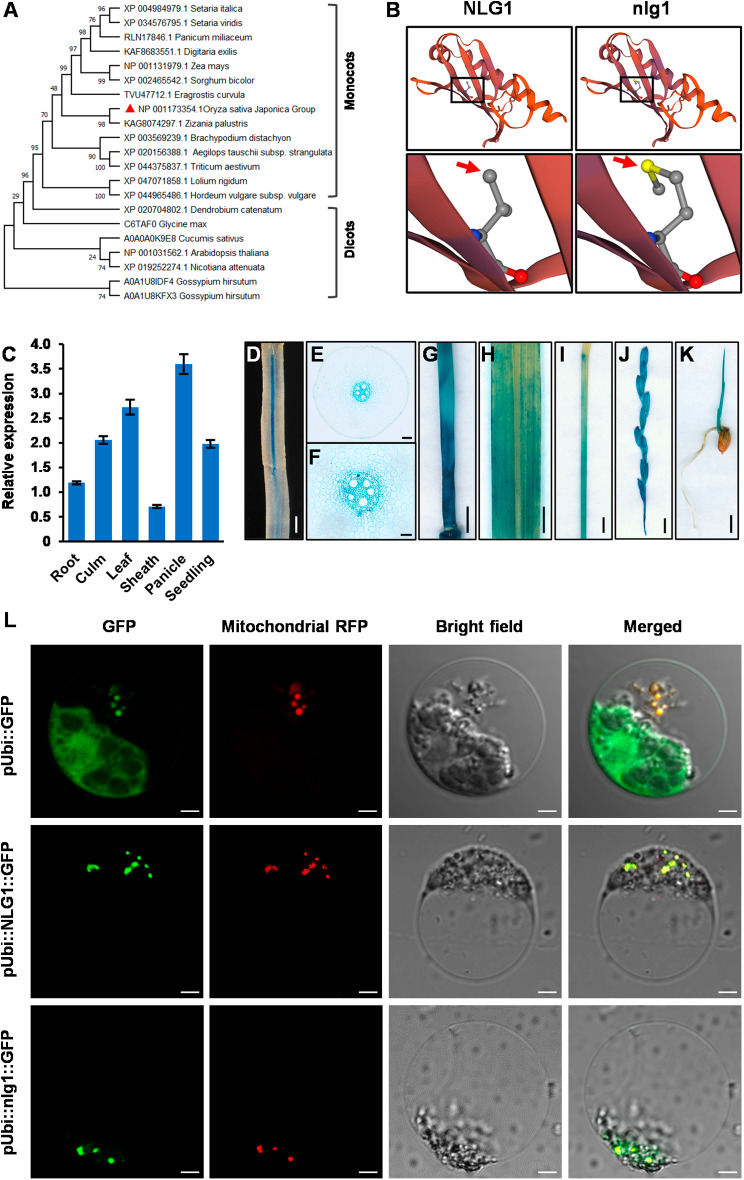
Expression pattern and subcellular localization of NLG1. **A** Phylogenetic tree showing evolutionary relationship among NLG1 and its homologs in other plants. Each protein was showed in species name and Genbank accession number. **B** Tertiary structure model of NLG1 and nlg1. The peptide bonds alteration was pointed out by red arrows. **C** Relative expression levels of *NLG1* in various organs from YD32 by RT-qPCR. Data represent means ± SD (*n* = 3). **D-K** Gus staining showed tissue specific expression of *NLG1*. **L** Subcellular localization of NLG1 and nlg1 in rice protoplasts. Mitochondrial RFP was used as a mitochondrial marker. Scale bars: 1 mm in **D**, 100 μm in **E**, 50 μm in **F**, 5 mm in **G-J**; 5 μm in **K**; 5 μm in **L**

Sequence analysis revealed that *NLG1* encodes a mitochondrial import inner membrane translocase Tim21. To further analyze the structure and function, NLG1 protein and 21 corresponding orthologs were used for phylogenetic analysis. The result indicated that NLG1 in rice has the closest evolutionary relation with its orthologous protein in *Zizania palustris*. Although SD3 (NP_001031562.1) is known as a Tim21 in *Arabidopsis*, it has a distinct evolutionary relationship with NLG1 (Fig. 5A) [36], revealing that the Tim21 functions may be differentiated in different species. Further protein alignment showed that these orthologs contained a highly conserved mitochondrial transmembrane region and Tim21 domain (Fig. S4A). Tertiary structure model analysis between NLG1 and nlg1 revealed that substitution of Met^227^ for Val^227^ in Tim21 domain caused a peptide bonds alteration, which may explain the disruptive function of *nlg1* (Fig. 5B).

### Expression pattern and subcellular localization of NLG1

To identify the spatio-temporal expression patterns of *NLG1*, we quantified the *NLG1* transcription level in various tissues and organs by RT-qPCR. The results suggested that *NLG1* was expressed abundantly in root, culm, leaf, leaf sheath, panicle and seedling, especially higher in young leaf and panicle (Fig. 5C). GUS staining assay were performed to determine the specific promoter activity of *NLG1*, and the staining tissues was consistent with RT-qPCR results. Moreover, the cross section of root showed a specific staining in vascular bundles (Fig. 5D-K). Therefore, *NLG1* functions in a constitutive expression pattern. To examine the subcellular localization of *NLG1*, the p*Ubi::NLG1::GFP* and p*Ubi::nlg1::GFP* fusion protein were expressed in rice protoplasts and the Mitochondria-RFP were used as mitochondria marker. The GFP signals were overlapped with Mitochondria-RFP signals, which manifested that both NLG1 and nlg1 were located in the mitochondria (Fig. 5L), and the mutation in *nlg1* did not affect the localization.

### Mitochondrial Structure Defection and Compromised ATP Content in *nlg1*

**Fig. 6:**
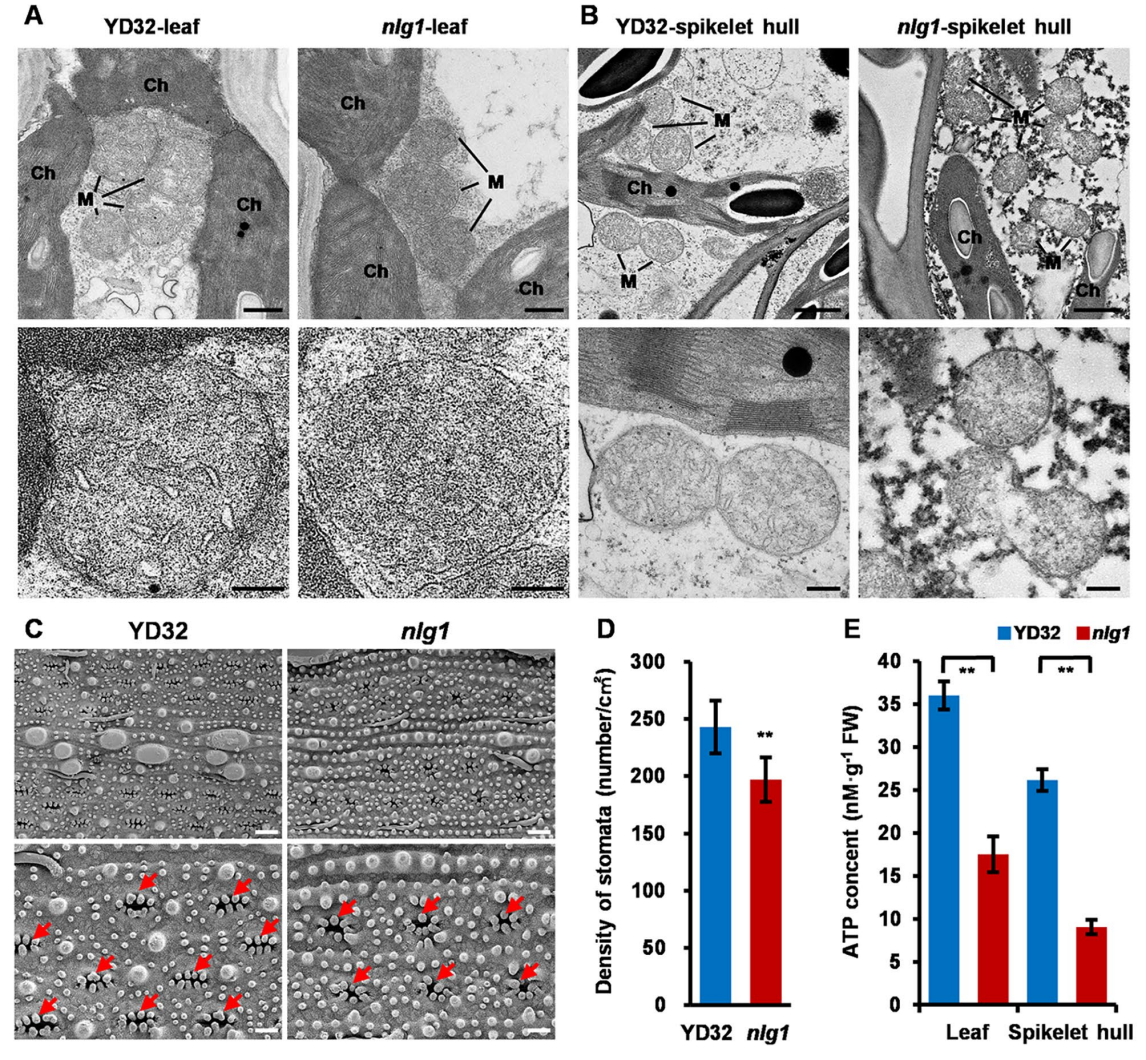
The mutation of NLG1 caused a mitochondria defection and stomata density reduction. **A-B** Ultrastructure of mitochondria in flag leaves (**A**) and spikelet hulls (**B**) from YD32 and *nlg1* observed by transmission electron microscopy. M, mitochondrial, Ch, chloroplast. **C** Scanning electron micrographs of leaf epidermal surface in flag leaves from YD32 and *nlg1*, red arrows indicate the stomata. **D** Density of stomata per cm^2^ in flag leaves from YD32 and *nlg1*. Data represent means ± SD (*n* = 10). **E** ATP content of flag leaves and spikelet hulls from YD32 and *nlg1* at heading stage. Data represent means ± SD (*n* = 6). **Significant difference at p < 0.01 compared with YD32 by Student’s *t*-test. Scale bars: 500 nm (up) and 200 nm (down) in **A**; 1 μm (up) and 250 nm (down) in **B**; 20 μm (up) and 10 μm (down) in **C**

To determine the effect of NLG1 on mitochondrial development, the TEM were conducted to observe the ultrastructure of mitochondria. Compared with the wild-type, the mitochondria in flag leaves and spikelet hulls of *nlg1* displayed abnormal and degraded cristae, which blurred the boundary of inner membranes (Fig. 6A-B). It is well known that mitochondrion is the site of plant oxidative respiration, organics decomposition and ATP production. So, we measured the ATP content of flag leaves and spikelet hulls in YD32 and *nlg1* at heading stage and found that the ATP content of *nlg1* was only half of YD32 (Fig. 6E). Moreover, we also observed the leaf epidermal cells via SEM. The results showed that the stomata density of *nlg1* was lower than YD32, which implied that aberrant mitochondria development may lead to a weaker respiration in *nlg1* (Fig. 6C-D).

Antimycin A and oligomycin are inhibitors of cytochrome c reductase and function in inhibiting electron transport from ubiquinone to cytochrome c, which may decrease ATP contents in mitochondria [46]. The 14-day-old seedlings of YD32 and *nlg1* were treated with antimycin A (1 µM) and oligomycin (0.2 µM) for 3 days, respectively, and survival rates were calculated to assess the sensitivity to antimycin A and oligomycin. The results showed that both YD32 and *nlg1* showed various degrees of withered leaves after treatment. However, *nlg1* mutants showed more severe growth defects and lower survival rate than that of YD32, indicating that *nlg1* were more sensitive to the inhibitors of mitochondrial electron transport chain (Fig. S5).

### *NLG1* influences auxin response and mitochondrial membrane development

**Fig. 7:**
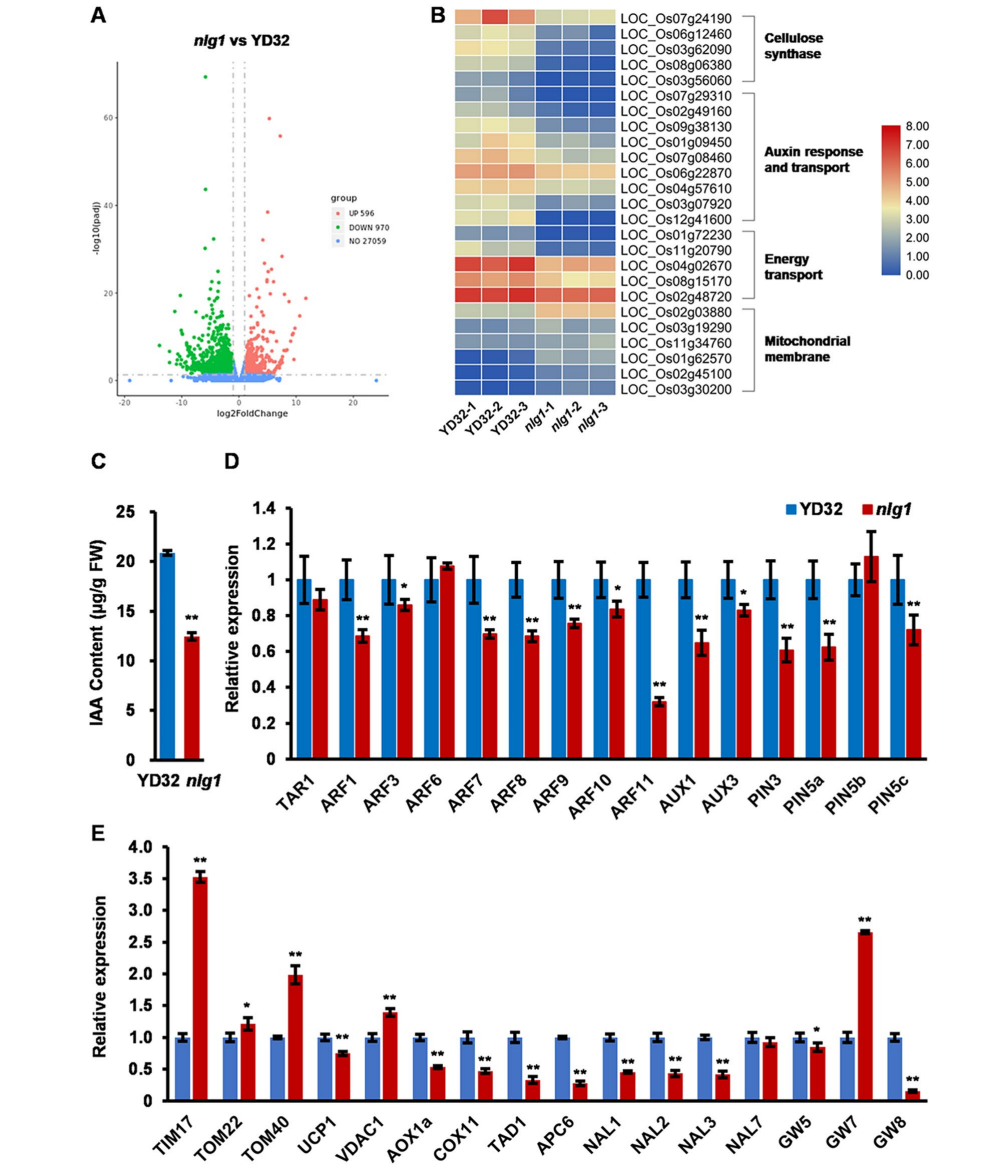
*NLG1* involved in auxin response pathway and mitochondrial membrane development. **A** Volcano plots of differentially expressed genes (DEGs) profile in *nlg1* mutant and wild-type YD32. **B** Heat map of cellulose synthase, auxin response and transport, energy transport and mitochondrial membrane translocase related DEGs expression. Data represent three biological replicates. **C** IAA content of leaf primordium at seedling stage in YD32 and *nlg1*. **D** Relative expression levels of genes involved in auxin biosynthesis, response and transport in flag leaves from YD32 and *nlg1* at reproductive stage. Data represent means ± SD (*n* = 3). **E** Relative expression level of genes involved in mitochondrial membrane development, respiratory chain complex, cell cycle, leaf width and grain width in flag leaves from YD32 and *nlg1* at reproductive stage. Data represent means ± SD (*n* = 3). **Significant difference at p < 0.01 and *difference at p < 0.05 compared with YD32 by Student’s *t*-test

To reveal the function of *NLG1* in regulating leaf width and grain size development, we conducted an RNA sequencing (RNA-seq) analysis. Compared with YD32, a total of 596 up and 970 down regulated differentially expressed genes (DEGs, threshold of twofold change ≥ 1 and p value ≤ 0.05) were detected in *nlg1* (Fig. 7A). These DEGs included many genes related to the mitochondrial membrane translocase (*LOC_Os03g19290* [*Tim17*], *LOC_Os02g45100* [*Tim23*] and *LOC_Os02g03880* [*TOM22*]), cellulose synthase (*LOC_Os07g24190*, *LOC_Os06g12460*, *LOC_Os08g06380*, *LOC_Os03g56060* and *LOC_Os03g62090*), auxin response and transport (*LOC_Os01g09450* [*IAA2*], *LOC_Os02g49160* [*IAA8*] and *LOC_Os04g57610*), and ATP synthase (*LOC_Os04g02670* and *LOC_Os08g15170*) (Fig. 7B, Table S3). These results suggested that NLG1 may participate in mitochondrial membrane development and ATP metabolism, regulating leaf width and grain size by affecting cellulose synthesis and auxin transport. Moreover, we further investigated the expression of a number of genes related to channel proteins located in mitochondrial membrane and respiratory chain complex. The results revealed that the expression level of translocase of the inner membrane gene *TIM17*, outer membrane genes *TOM40* and *TOM22* were significantly up-regulated, and membrane channel protein genes *UCP1* and *VDAC1*were obviously changed in *nlg1*, while the respiratory function related genes *AOX1a* and *COX11* were down-regulated markedly in *nlg1* compared with YD32 (Fig. 7E).

Narrow leaf and slender grain are generally associated with the synthesis and transduction of auxin signal. The auxin biosynthesis mutant *fib*, polar auxin transporter (PAT) mutant *nal1* and auxin responder mutant *nal21* were all presented narrow leaves and small panicles [5, 11, 47]. Considering phenotypic similarity of *nlg1* with *fib*, *nal1* and *nal21*, the internal IAA levels of the leaf primordium from YD32 and *nlg1* were measured. As expected, the internal IAA content were 40.3% reduction in *nlg1* than YD32 (Fig. 7C). We further detected the expression of auxin biosynthesis, response and transport genes, and found that auxin response genes *ARF1*, *ARF3*, *ARF7*, *ARF8, ARF9*, *ARF10* and *ARF11*, auxin influx carrier genes *AUX1* and *AUX3*, auxin efflux carrier genes *PIN3*, *PIN5a* and *PIN5c* were significantly down-regulated in *nlg1*, which is consistent with the RNA-seq consequence (Fig. 7D). Meanwhile, the expression levels of narrow leaf, grain width and cell cycle genes were also detected. The results showed that the narrow leaf genes *NAL1*, *NAL2* and *NAL3*, grain width genes *GW5* and *GW8*, cell cycle-related genes *TAD1* and *APC6* were markedly down-regulated, while *GW7* expression was highly up-regulated in *nlg1* than that of YD32 (Fig. 7E), indicating that disrupted expression of these genes may be responsible for the narrow leaf and slender grain phenotype of *nlg1* mutant.

## Discussion

### NLG1 encodes a mitochondrial Tim21 affecting leaf shape and grain size

Mitochondrion is a double-layer membrane organelle that exists in most eukaryotic cells, providing the main site for aerobic respiration. Mitochondrial membrane system comprises smooth outer membrane and wrinkled inner membrane. The latter possess a sophisticated topology, which is highly folded to form cristae to increase functional area [48]. The preprotein translocase of inner membrane TIM23 complex and translocase of outer membrane TOM complex mainly acts in protein transport [49]. Among them, Tim21 is an essential component of TIM23 complex that mediates the translocation of transit peptide-containing proteins sort into the mitochondrial inner membrane with the cooperation of TOM complex. In *S. cerevisiae*, Tim21 interacts with Tim23 and Tim50, and also interacts with Tom22 of TOM complex to maintain the close touch of TIM23 and TOM complex. Under a certain circumstance, Tim21 is released from the TIM23 complex and directs the translocation of preprotein into mitochondrial matrix [50–52]. In *Arabidopsis*, it has been reported that Tim21 (SD3) act as a coordinator in retrograde signaling from mitochondrion to nucleus. *sd3* mutant shows dwarf and seedling lethality, and decreased intracellular ATP content [36]. However, the function of Tim21 mitochondrial protein has not been reported in rice. In this study, we identified a narrow leaf and slender grain mutant *nlg1*, which encodes a mitochondrial import inner membrane translocase Tim21 and located in mitochondria (Figs. 1, 2, 3, 4 and 5). Similar to *sd3* in Arabidopsis, *nlg1* showed a dwarf and weak growth phenotype, indicating that NLG1 has similar functions to those in Arabidopsis and is essential to maintain the plant vegetative growth. However, *nlg1* also exhibited narrow leaves and slender grains, and the narrow leaf phenotype of *nlg1* can be clearly distinguished just 10 days after germination (Fig. S1), suggesting that the functions of NLG1 and SD3 are not totally identical. Phylogenetic analysis showed that the homologous proteins of NLG1 are highly conserved in monocots and significantly differentiated from dicots (Fig. 5A and S4), implying that NLG1 may obtain additional functions to regulate leaf and grain development.

### NLG1 involved in the construction of mitochondrion and determination of respiratory chain function

The formation of mitochondria cristae and the biogenesis of respiratory chain relies on the presequence-containing protein import and proper assembly, which required for a precise mitochondrial import membrane system. TOM and TIM complex in outer and inner membrane is the main import gate [53, 54]. Among them, Tim21 anchors in the inner membrane via a transmembrane domain and specifically interacts with TOM complex by C-terminal domain to keep closely connection between TIM and TOM [50, 55]. We noticed that the *nlg1* showed abnormal mitochondrial development with blurry cristae boundary and declined intracellular ATP content than YD32 (Fig. 6A-B), suggesting that the mutation of *NLG1* disrupted the structure of mitochondrion and the synthesis of ATP.

It has been reported that the mitochondrial respiratory chain assembled with imported peptides and mitochondrial-synthesized peptides, which is coordinated via multiple mechanisms occurred in mitochondrial outer membrane, inner membrane and matrix [56]. As a component of mitochondrial TIM23 complex translocase, Tim21 interacts with respiratory chain complexes and also mediates the assembly of preprotein into intermediates after import into mitochondria in the formation of respiratory chain [57, 58]. We treated YD32 and *nlg1* plants with respiratory chain inhibitor antimycin A and ATP synthesis blocker oligomycin, and found the survival rate of *nlg1* was significantly lower than that of wild-type YD32. The result revealed that the defect of mitochondria in *nlg1* results in a sensitivity to antimycin A and oligomycin, implying that respiratory chain is disordered (Fig. S5). Besides, the expression of several genes related to mitochondrial membrane protein such as *TIM17*, *TOM40*, *TOM22* and *VDAC1* were raised significantly, and respiratory-chain function genes *AOX1a* and *COX11* were clearly repressed in *nlg1*, revealing that the loss function of *NLG1* may lead to a disruption of membrane system and electron transport chain in mitochondria (Fig. 7E). Therefore, we speculate that NLG1 controls plant growth through coordinated the expression of the respiratory complex genes, and is required for the determination of mitochondrial membrane morphology and respiratory chain function.

### NLG1 may regulates leaf and grain growth by mediating auxin response

Many studies have shown that auxin plays a vital role in regulating leaf primordium differentiation and cell proliferation, and the auxin deficiency is closely related to narrow leaf phenotype [5, 6, 9, 11, 59, 60]. Mitochondria and auxin act as the metabolic homeostasis maintainer and signal conductor in plant growth and development [61]. More and more evidence proved that they are interconnected. In fact, mitochondrial perturbation negatively affects auxin signaling, in turn, auxin signaling networks control mitochondrial metabolic and energy pathways in cellular function and plant growth [62, 63]. In this study, the *nlg1* also exhibited dwarfism and narrow leaves similar to the mutant of auxin deficiency (Fig. 1). *DVB1* encodes a Mic10 family protein, may be required for the connection between the mitochondria development and IAA synthesis. The *dvb1* mutant exhibits a narrow leaf and abnormal mitochondria structure phenotype similar to *nlg1* [16]. In addition, our results showed that the expression level of auxin response and efflux carrier related genes such as *ARFs* and *PINs* were down-regulated significantly, and IAA content were also decreased in the *nlg1* leaves compared with YD32. RNA-seq analysis also revealed that many DEGs related to the auxin response and transport were remarkably changed between *nlg1* and YD32, which further suggesting that *NLG1* may regulates leaf and grain development by mediating auxin response (Fig. 7). However, the sufficient and specific evidence is still lacked on how NLG1 affects auxin response to regulate leaf width and grain size, and sustain mitochondrion development. Further study will be needed to elucidate the role of NLG1 in the connection between mitochondria and auxin response.

### Electronic supplementary material

Below is the link to the electronic supplementary material.

Supplementary Material 1

Supplementary Material 2

Supplementary Material 3

Supplementary Material 4

Supplementary Material 5

Supplementary Material 6

Supplementary Material 7

Supplementary Material 8

Supplementary Material 9

Supplementary Material 10

## Data Availability

The original contributions presented in the study are included in the article and Supplementary materials, further inquiries can be directed to the corresponding author. The raw data of RNA-seq generated during the current study are available in the Sequencing Read Archive (SRA) of NCBI (PRJNA940852).
