# Supplementary material for: NLG1, encoding a mitochondrial membrane protein, controls leaf and grain development in rice
Source: BMC Plant Biol. 2023 Sep 9;23:418. doi: 10.1186/s12870-023-04417-2 (PMC10492415; doi:10.1186/s12870-023-04417-2)
Supplement: Supplementary file 1 — Supplementary Material 1 [file 12870_2023_4417_MOESM1_ESM.docx]

Additional file 6

Table S1. Annotation of the 6 ORFs in the 42.1-kb target region

| ORFs | Annotation |
| --- | --- |
| LOC_Os03g14840 | AGC_PVPK_like_kin82y.8 - ACG kinases include homologs to PKA, PKG and PKC, expressed |
| LOC_Os03g14850 | OsMADS72 - MADS-box family gene with M-alpha type-box, expressed |
| LOC_Os03g14860 | G-patch domain containing protein, expressed |
| LOC_Os03g14880 | expressed protein |
| LOC_Os03g14890 | AGAP008572-PA, putative, expressed |
| LOC_Os03g14900 | NB-ARC/LRR disease resistance protein, putative, expressed |
|  |  |
|  |  |
