# Supplementary material for: NLG1, encoding a mitochondrial membrane protein, controls leaf and grain development in rice
Source: BMC Plant Biol. 2023 Sep 9;23:418. doi: 10.1186/s12870-023-04417-2 (PMC10492415; doi:10.1186/s12870-023-04417-2)
Supplement: Supplementary file 2 — Supplementary Material 2 [file 12870_2023_4417_MOESM2_ESM.docx]

Additional file 7

Table S2. Primers used in this study

| Name | Sequence |
| --- | --- |
| **Gene mapping** | |
| M34-F | CAGCTGAGAAGCCATCC |
| M34-R | CACATGACAGCCAGTCGG |
|  |  |
| M35-F | TGGCCGTTGAGGCGATTAG |
| M35-R | CAGAGTCTTACTGCTAACCCCGT |
|  |  |
| M36-F | TCCTGTGTTCGCCTGCTCGTC |
| M36-R | TTCGTGGTCGCTGAAAAGTCC |
|  |  |
| M37-F | GCGCGATAAACGTTTGAGAGAAGG |
| M37-R | AAATCAGTCGCGGTTGCTGTCC |
|  |  |
| L8-F | GTGTTTCACGTTGCGTACGTGCG |
| L8-R | CGTTCACCGATCTGCTCTCCTCG |
|  |  |
| L46-F | GTTGCGTTGAAATGTTGCTATG |
| L46-R | GTACTCCCTCCTCATATTACAGAC |
|  |  |
| L53-F | GACACGAGGTAGGCATGAGAC |
| L53-R | CACGAGAAGAAATGATGCAAAC |
|  |  |
| L62-F | TCTAAACGTGACTGAAAGGAGGC |
| L62-R | TGACTCATCTTCTATTAGTGG |
|  |  |
| L56-F | GGCTCGGCCAACCAATTGGGC |
| L56-R | CGCACGATGGATCCGGCTCAC |
|  |  |
| L24-F | GTAGTCCTGGTTGTATCCGAG |
| L24-R | ACTACCAATCGAGACCTGGTCTC |
|  |  |
| L57-F | GATTCAAGGGGTAGGTAGGTTG |
| L57-R | CTTCCTTTCTCTCCTAGGTCCAC |
|  |  |
| L41-F | ATGTCACATCCTAAGTTGAG |
| L41-R | TTATTTAATTATATCGTAATGCCT |
|  |  |
| L35-F | CAGGCCAAGTGCAGCATCGATC |
| L35-R | GCACCGTTTAGTTGTTTA |
|  |  |
| L39-F | GAGTTCGTATGGGTTACTTA |
| L39-R | GCCTCGGTTCGTGCTGCATG |
|  |  |
| 14890-1F | ATATAAGGCATACATGCATAC |
| 14890-1R | CACAGCAATGTTCTGCAACTA |
|  |  |
| 14890-2F | GCGCAGCCCCTACCATCACTG |
| 14890-2R | GTTACAACCTCCCCGGTTGCC |
|  |  |
| 14890-3F | ATGGCGTCCCGGATCGCGAGG |
| 14890-3R | GAGTAAATAGCATACTATCAT |
|  |  |
| 14890-4F | CATAGTATGACAGAGAGTTGC |
| 14890-4R | CTCCAGCAATACGTTGCCAACTG |
|  |  |
| 14890-5F | CAATCTAAGCTGGGAGGCTGC |
| 14890-5R | GCTAAGTGACTTGCATAGGATC |
|  |  |
| 14890-6F | TATGACCCTCACATGTAGCCAC |
| 14890-6R | GTGCACATACGTCAACTGTATG |
|  |  |
| 14890-7F | CATGGACACATGCTCCTTGAC |
| 14890-7R | TAAGTAAAAGAATTATGAAAGGTTATCG |
|  |  |
| 14890-8F | TATTAGTGAGAATGGACCA |
| 14890-8R | GCTGGAGGCTGTATCAGGTG |
|  |  |
| **Vector construction** | |
| NLG1-OE-F | CTTCTGCAGGAGCTCGATGGCGTCCCGGATCGCG |
| NLG1-OE-R | TGCTCACCATGGATCCTGCTGGTACGTAAGATTC |
|  |  |
| NLG1-1305GUS-F | GCAGGCATGCAAGCTTATCCAAACTATGAGGTGA |
| NLG1-1305GUS-R | CTCAGATCTACCATGGTGAGGGGGGGAGTCTCTC |
|  |  |
| NLG1-COM1-F | TCGACCTGCAGGCATGCAATCCAAACTATGAGGTGA |
| NLG1-COM1-R | CGACGGCCAGTGCCAAGCTTACAAGATTCACTAATGTCT |
|  |  |
| NLG1-COM2-F | AGACATTAGTGAATCTTGTGTTGTTGGCTTCTCTAAC |
| NLG1-COM2-R | CGACGGCCAGTGCCAAGCTTAAGTAAAAGAATTATGA |
|  |  |
| NLG1-RNAi-1F | CGATCGGGGAAATTCGAGTCAAACCCTTCAAGTCCTC |
| NLG1-RNAi-1R | GATTTTCAGATCGATACTCTTGTACTCTTTTGGTTC |
|  |  |
| NLG1-RNAi-2F | TCTGTCGACCTCGAGGGTCTTGTACTCTTTTGGTTC |
| NLG1-RNAi-2R | CAGGTCGACTCTAGAGGATCAAACCCTTCAAGTCCT |
|  |  |
| **RT-qPCR** | |
| TIM17-RT-F | GCTGCTGGTGGTGACCGCAAGT |
| TIM17-RT-R | TCCCTGTAGTCGAAGGATGGAA |
|  |  |
| TOM22-RT-F | GGCGAGCAGGGCGAAGAAGGTG |
| TOM22-RT-R | CGATGATGAGCGGGACGACGAG |
|  |  |
| TOM40-RT-F | GTGACAATCCTTGTGCGCC |
| TOM40-RT-R | GAACTCGTAGTTGGAGGTGG |
|  |  |
| VDAC1-RT-F | TTCCAAGAGTACAACACCTGTC |
| VDAC1-RT-R | GGCTCGGATGAAGAGTATTTAG |
|  |  |
| AOX1a-RT-F | TGCGGCTGATGTCCACGTC |
| AOX1a-RT-R | GTTGACCACCACCTCCTTC |
|  |  |
| COX11-RT-F | GAGGAGCAAACACTTCTTCCAG |
| COX11-RT-R | TTCCATTTTGGGGTCTGTCTCA |
|  |  |
| UCP1-RT-F | TTTGGAACGGCATCATCCC |
| UCP1-RT-R | GCTCCAATACCTTCCTGTC |
|  |  |
| YUCCA1-RT-F | CGCAACACGGTGCATGTC |
| YUCCA1-RT-R | CGATGCCGAACGTGGATAG |
|  |  |
| TAR1-RT-F | GAGGTGCGCCGCCTCCA |
| TAR1-RT-R | GAGCGCGGCCTGGAAGAG |
|  |  |
| ARF1-RT-F | GCACTCCTTCTGCAAGATCC |
| ARF1-RT-R | GGCCCCTGTAGATGTGCTTA |
|  |  |
| ARF3-RT-F | TCTCGTCCCTCTACGTGCTT |
| ARF3-RT-R | CCGATTACAACGGGAGCTTA |
|  |  |
| ARF6-RT-F | CAAGAGTTGATGGCGAAAGACC |
| ARF6-RT-R | CAACCCGTAGTCAGAAGATGCC |
|  |  |
| ARF7-RT-F | CTTGACCAGCATCTGCCTTTGT |
| ARF7-RT-R | ATCTGAATCGGTTTCAGCCCTA |
|  |  |
| ARF8-RT-F | AGATGAGCCTTAGCGGCACCAC |
| ARF8-RT-R | CCGAGCCCTCCGAGGCACCTTT |
|  |  |
| ARF9-RT-F | ATGGCCGCCGCGATGGAGATGGCGG |
| ARF9-RT-R | CCGCCATCTCCATCGCGGCGGCCAT |
|  |  |
| ARF10-RT-F | GCAGCCGAATCTGATGCTCTAC |
| ARF10-RT-R | TCGGTGCTGATGATGATTCTCG |
|  |  |
| ARF12-RT-F | CTTGGGAGTCCTTTGTGAATAG |
| ARF12-RT-R | GACAGATACCGTGGATCATTTC |
|  |  |
| AUX1-RT-F | TCGCCCACATCCTCACCTACCG |
| AUX1-RT-R | GAGCACGAACATCCCCGTCCAG |
|  |  |
| AUX3-RT-F | CAGGTGTTCTACGGCCTCATGG |
| AUX3-RT-R | CTGACGCCTTCCTTCTCCTTGC |
|  |  |
| PIN3-RT-F | TTGTTCAGGCGGCTCTACCA |
| PIN3-RT-R | AAGGAAATTGCTTACGCTGT |
|  |  |
| PIN5a-RT-F | ACCTGGGCCTGCCTCGCCAATA |
| PIN5a-RT-R | GCCATTCCTGTGCCTGACTTGG |
|  |  |
| PIN5b-RT-F | GGTCGGTGAGGTGGTGGAGGTT |
| PIN5b-RT-R | AGGTGAAGAAGGGCATGGAGAA |
|  |  |
| PIN5c-RT-F | CCTCAATCCATCACGACGTTCG |
| PIN5c-RT-R | TAATGACGCCAGTGTCCCGAAT |
|  |  |
| TAD1-RT-F | GTAACTGGCGCTGGTGATGAAA |
| TAD1-RT-R | CCCCGATGCTACTTAGGCTGTC |
|  |  |
| APC6-RT-F | GGCATACGAAGCACTGGACAAC |
| APC6-RT-R | TTCAAGGGCCTCATAGCACAAA |
|  |  |
| NAL1-RT-F | CCCTGGTCGGTTGCCCAAAGGT |
| NAL1-RT-R | TGCGGAATCCCACTGCTGTCCC |
|  |  |
| NAL2-RT-F | GGTCGAGTCGCTAGGAGGGAG |
| NAL2-RT-R | CTACCTCTCTCCCAATTCTCTC |
|  |  |
| NAL3-RT-F | GCCGCATCGAGGGCAAGAACGT |
| NAL3-RT-R | GTGGAGGAGGGCGGTGAGGGTT |
|  |  |
| NAL7-RT-F | GGTGAGTTGTTCACGGAGGAGG |
| NAL7-RT-R | CGGAGAATCCCACGCAGTAGAG |
|  |  |
| GW5-RT-F | CACTCGTCGCCGCTCAACTGCC |
| GW5-RT-R | TCTGCGAACGCACCTTCGCCTC |
|  |  |
| GW7-RT-F | GCTGCCAAAAGATGAGTCTCCG |
| GW7-RT-R | CAATCTTCTCCTGCTGCCTCCA |
|  |  |
| GW8-RT-F | TTCTGCCAGCAGTGCAGCAGGTT |
| GW8-RT-R | GGCGACGGTTGTGCCCATCTAG |
