# Supplementary material for: NLG1, encoding a mitochondrial membrane protein, controls leaf and grain development in rice
Source: BMC Plant Biol. 2023 Sep 9;23:418. doi: 10.1186/s12870-023-04417-2 (PMC10492415; doi:10.1186/s12870-023-04417-2)
Supplement: Supplementary file 3 — Supplementary Material 3 [file 12870_2023_4417_MOESM3_ESM.docx]

>NP_001173354.1 probable mitochondrial import inner membrane translocase subunit TIM21 [Oryza sativa Japonica Group]

MASRIARLLQHQNRRLLSTAAEASSRRPPRAPLSGAIPKHEVAKAEASSLKKSRWYMIKSNPSSPLTTQRESHKVSTHLVRPSASYSTQASEQNPKEGTKDLKTVEDPFDSPTYNIPEKPVTFAEGASYSLVIVAGLGIAAVAGYAVFKELIFEPKEYKIFGKALARVQNDSQVTAKIGYPVTGYGNESRNRAARQRIPNKVWTDEDGVEHVEVNFYIRGPHGAGKVYSEMFKDNNDRSWKFTYLIVEIVSPHRVQLMLESYVPA

>XP_004984979.1 probable mitochondrial import inner membrane translocase subunit TIM21 [Setaria italica]

MASRIARLLQHRRLLATAAEASARHAPRAPCAGAAISKDVAKAEASSLKNSRWYMTRSNTSGPLTTHSECRKAFPSFMRPSASYSTKASDQNPKQEGKDLSTTEHPFDDITYNIPEKPVTFTEGASYSLVILAGLGIAALAGYAVFKELIFEPKEYKIFGKALARIQSDSQVTARIGHPITGYGQETRNRAARQRIPNKIWTDEDGVEHVEVNFLIRGPHGAGKVYSEMFKDNSDRTWKFTYLVVEIVSPQHGKTQLMLESYLPA

>XP_034576795.1 probable mitochondrial import inner membrane translocase subunit TIM21 [Setaria viridis]

MASWIARLLQHRRLLATAAEASARHAPRAPCAGASISKDVAKAEASSLKNSRWYMTRSNTSGPLTTHSECRKAFPSFMRPSASYSTKASDQNPKQEGKDLSTTEHPFDDITYNIPEKPVTFTEGASYSLVILAGLGIAALAGYAVFKELIFEPKEYKIFGKALARIQSDSQVTARIGHPITGYGQETRNRAARQRIPNKIWTDEDGVEHVEVNFLIRGPHGAGKVYSEMFKDNSDRTWKFTYLVVEIVSPQHGKTQLMLESYLPA

>RLN17846.1 putative mitochondrial import inner membrane translocase subunit TIM21 [Panicum miliaceum]

MASRMARLLQHRRLLATAAEASARHAPRAPCAGAAVSKDVAKAEALSLKNSRWYMTRSNTSGPLTSHYECRKAFPSFIRPSASYSTKASDQNPKQEGKDLSTTEHPFDDITYNIPEKPVTFTEGASYSLVILAGLGIAALAGYAVFKELIFEPKEYKIFGKALARIQSDSQVTARIGHPITGYGQETRNRAARQRIPNKIWTDEDGVEHVEVNFLIRGPHGAGKVYSEMFKDNSDRTWKFTYLVVEIVSPQHAKTQLMLESYLPA

>KAF8683551.1 hypothetical protein HU200_044466 [Digitaria exilis]

MASRIARLLQHRRLLATAAEASARHAPRAPCAGAAISKSWGKYPASSLKNSRWYMTRSNTSGSLTTHYECRKAFPSFIRPSASYSTEASNQNPKQEGKDLSTTEHPFDDITYNIPEKPVTFTEGASYSLVILAGLGIAALAGYAVFKELIFEPKEYKIFGKALARIQSDSQVTSRIGHPITGYGQETRNRAARQRIPNKIWTDEDGVEHVEVNFLIRGPHGAGKVYSEMFKDNSDRTWKFTYLVVEIVSPQHAKTQLMLESYLPA

>XP_020156388.1 probable mitochondrial import inner membrane translocase subunit TIM21 [Aegilops tauschii subsp. strangulata]

MAALITRLLHHHNCRLLATAAEASARRLPRAPLGGAIYKDVAKAEASSLKNSRWYMTKSSPFSPLTMQEYRKTFPSLLRSSASYSSQASDQNPKEEKKDLSTVGDPFDAPTYNIPEKPVTFAEGASYSVVILAGLGVAALAGYSVFKELIFEPKEYKIFGKALARVQSDSQVTARIGYPITGYGNESRNRAARQRIPNRVWTDEDGVEHVEVNFYIRGPHGAGKVYSEMFKDNSDRTWKFMYLLVEFTASLQGQVMLESYIPA

>XP_044375837.1 probable mitochondrial import inner membrane translocase subunit TIM21 [Triticum aestivum]

MAALITRLLHHHNRRLLASARRLPRAPLGGAIYKDVAKAEASSLKNSRWYMTKSSPFSPLTMQEYRKTFPSLLRSSASYSSQASDQNPKEEKKDLSTVGDPFDAPTYNIPEKPVTFAEGASYSVVILAGLGVAALAGYSVFKELIFEPKEYKIFGKALARVQSDSQVTARIGYPITGYGNESRNRAARQRIPNRVWTDEDGVEHVEVNFYIRGPHGAGKVYSEMFKDNSDRTWKFMYLLVEFTAPLQGQVMLESYIPA

>NP_001131979.1 Probable mitochondrial import inner membrane translocase subunit TIM21-like [Zea mays]

MASRVTRLLLHRRLTTAAEASARRAPQAPCAGAAVSKDVVKAEASSLKNSRWHITRSNTSGPLTTRYECRKVFPCSVRPSASYSTQASDQKGKQEGKDLSNVEHPFDDITYNIPEKPVTFTEGASYSLVILAGLGIAGLAGYAVFKELIFEPKEYKIFGKALARIQSDSQVTSRIGHPITGYGQETRNRAARQRIPNKIWTDEDGVEHVEVNFLIRGPHGAGKVYSEMFKDTDRTWKFTYLVVDIVSPPHAKTQLMLESYLPA

>XP_003569239.1 probable mitochondrial import inner membrane translocase subunit TIM21 isoform X1 [Brachypodium distachyon]

MAWRRVLSQAARNQSAYAIYNELAAPSPLRSLRSNISAGVTLRNLHERYYSSYFGSLSRSARDLGSPSEASLLKEIYRSDPERVIQIFEGQPSLHSNPSALSEYVKALVKVDRLDESILLKTLQRGFSLKNSRWYMIKSSPSNPLTMRECHKVFPSLIRPSASYSTQASDKKTQKERKDLSTTEDPFDDAPTYNIPEKPVTFAEGASYSLVILAGLGVAALAGYAVFKELIFEPKEYKIFGKALARVQSDSQVTARIGYPITGYGSETRNRAARQRVPSRIWTDEEGVEHVEVNFYIRGPHGAGKVYSAMFKDNSDGAWKFTYLLVEFTAPHQGQVMLESYIPA

>XP_047071858.1 probable mitochondrial import inner membrane translocase subunit TIM21 [Lolium rigidum]

MAAAAARAGSRRLFSTSALVTRKLLTPPAKADALPNLFVPGLGKQTAIGGLREAFLKSGQNAHASSLQNSRWYMISSNRSGSLAVRKEYGKVLPSSIRPCASYSTQASENKPKQEKTDLTTTEDPFDAPTYNIPEKPVTFAEGASYSVVILAGLGVAAMAGYAVFKELIFEPKEYKIFGKALARVQSDSQVTAKIGYPVTGYGTESRNRAARQRIQNRVWTDEDGVEHVEVAFFIRGPHGAGKVFAEMFKDDSDRTWKFTFLLVEVTSPRPAQIMLESYIPA

>XP_002465542.1 probable mitochondrial import inner membrane translocase subunit TIM21 [Sorghum bicolor]

MKATLHLLLRPPLALGMKATEAALRLVLSATAGQSQARRQCQRLLPPLASRLAPSPPCSAHRPATLLSSLKNSRWYVTRSNTSAPLTTRYEWRKVFPCSVRPCASYSTQADQKAKQEGKDLSTVEHPFDDITYNIPEKPVTFTEGASYSLVILAGLGIAGVAGYAVFKELIFEPKEYKIFGKALARIQSDSQVTSRIGHPITGYGQETRNRAARQRIPNKIWTDEDGVEHVEVNFLIRGPHGAGKVYSEMFKDNSDRTWKFTYLVVDIVSPPHAKTQLMLESYLPA

>NP_001031562.1 SD3 Arabidopsis thaliana

MMMMNLLRRSAIAIGRQSKSKLASFSSATQPCSGIPKSSKRVFSNSFLSKDSTGANGLLFRFRNPQASICTEARPKNINSSYFTRSFASRTSKEPGNQQNKAKKEVTTVEDPFDSPTYHIPEKPVTFTEGASYSLVILAGLGVAGAAGYGVFKELIFQPKEYKVFDKALKRIQDDGQVRVRIGSPIKGYGQETRNRAARQRIPNRVFTDEDGVEHVEVNFYIRGPQGAGKVYTEMFKDKAEKEWKYTYLIVEILTPSPAKLMLESYLPA

>AT2G40800 Arabidopsis thaliana

MVKSDKAGRHYTRVNVTGRSNSASSASNGVSKSHSSSKTSKNVGYSSKNKAGRVNVSASARVSTKSSGRKVDGSARKVVDKVKAVSSTARYRAGHDAWKKNSVVGAAGVVCWRMGASTVGSGMAKYGASSAVASGYRSRTNDKVYRMTMRKNTAAVMGASGSDRAYVMSGGGTKKKTRSKRCVGSKGVSVVKKKKGVSSSSSTSSVM

>AT3G56430 Arabidopsis thaliana

MVKSDKAVHKHYSRVNTTTGWSKSASSASNGVSKSGHTSSRTSKNGSSNGVSKTSHSSRTSKNVGSSKVGNCGVAVKRVNMNVSARSSSGRKVDGNARKVVDKKAVSSTARYRMAGHVDAWKKNNVGAGAVVCWRVMGASTVGSGMAKYGASSAVAAGYRARTNDKVYRTMRKNTAADVVMGAAGSDRAYVMSGGGTKKKTRNKRCVGSRKGVSVVKKKKGYDMKAVDMASGDRGDYRVGGGSRDVVKAMAATKDNDRDARARKRARKRARKRKK

>KAG8074297.1 hypothetical protein GUJ93_ZPchr0006g45783 [Zizania palustris]

MASRITRLLHHQRRRLLSTAAEASSRRPPRAPLGGAVSKHDAAKAEASYLLSSRQYMIKSNPSNPLTAQYDCHKVFSSLVRHSASYSTQASDQNPKEGRKDLSTVEDPFDAPTYNIPEKPVTFAEGASYSFVILAGLGIAAVAGYAVFKELIFEPKEYKIFGKALARVQNESQVTAKIGYPITGYGQETRNRAARQRIPNKVWTDEDGVEHVEVNFYIRGPHGAGKLLEAFSNHPQIKEVPETDRFLVRFAESDQHPRRTRRRKRGVVTGEMLAPLLGAGRSAWTGQDGGNAVTRQILKCTRWQLEETTDFITCPYHYSCDSDYPGDYHAAVGVLVAAFAAYCFISTLVFTVLELARSNAAGIRGIKRKYLLPSGPFLLPLVLLALAKGQRINAVFPLAQLGPALLLLLQASALAFRNEADGDIRYAVLEASTVSGVLHASLYLDAVLLPYYTGLEALRWSQFSGECASCLCRMEPLVVGGTTVRYRGLSKTALAIIFAMCSRMVCRIYGEDWLSAWTRSALECAGWVFVAADAVYLVGWVAADGGAVGVLAYSLVAGLVFLSVFGKVYRFLAWVETRQSQRKSNLCHSVV

>TVU47712.1 hypothetical protein EJB05_07319, partial [Eragrostis curvula]

MASRITRLLHHQHRRALATAAEAAARHAPRGPSAASLAKDVAKAEASSVKNSRWFMIRSNPSGSLTTRYECRRVSPSLIRPSASYSTRASDKNPKEARKDLSAVEEEPFDAITDKIPEKPVTFAEGASYSLVILAGLGIAAVAGYAVFKELIFEPKEYKIFGKALARIQSDSQIAARIGHPITGYGHETRNRAARQRITNKVWTDEDGVEHVEVNFLIRGPHGTGKVYSEMFKDNSDRTWKFTYLLVDIVSPHPQRLMLESYVPSYAPA

>XP_019252274.1 PREDICTED: probable mitochondrial import inner membrane translocase subunit TIM21 [Nicotiana attenuata]

MQHVRRSGVSLKTRWWSNVVKSSNKFDSLLEYMPSRSLSDLGISRFSSMAAETAAFNVSVKRELVRSGCARRHASYPAAKVIDEPAGLPVLYRNRRSTQCLMTFRSSFQLVNTAGVNPESSCFARSFASKASGSTQKQSEARKDVSTVEDPFDAPTYNIPEKPVTFTEGASYSLVILAGLAVAAGAAYGVFKELIFQPKEYKIFDKALKRIQNDGQVSVRIGSPVTGYGSESRNRAARQRIPNRIWTDEDGVEHVEVNFYIRGPHGAGKVYTEMFQDKVDRQWKFTYLIVEIKSPSPAQLMLESYVPA

>XP_020704802.1 probable mitochondrial import inner membrane translocase subunit TIM21 [Dendrobium catenatum]

MHQFKSKVRELGYLTQYKAFFSSSWGLSSAGLYPESSRRFEPLKRVVVISGYGSCCTSTTRRIVKDITKA

EGSSVPFAELFSRYISSIPSKQLNINDTKHCMLPSFTRPLSSNYSSQSSQQAKEESRKDISTIEDPFDDA

PTYNIPEKPVTFVEGASYGVIILAGLGVAALAAYAVFKELIFEPKEYKIFGKALDRVQNDSQVKVRIGSP

VTGYGQESRNRAARQRISNRVWKDEDGVEHVEVNFYIRGPHGAGKVFAEMFKDNSDKQWRFTYLIVEIMSPTHTQLMLESYVPA

>XP_044965486.1 probable mitochondrial import inner membrane translocase subunit TIM21 [Hordeum vulgare subsp. vulgare]

MAAAAARSGSRRLFSISALVPPKPPTPPPKADPFASLFIPGLSKRTTTDGLREAFAKSGEVVHASSVKSS

RWYMISANRSGPLTVRKEYRKVLPSFIRPSASYSTKASEKRPKQERTDLTTVEDPFNAPTYNIPEKPVTF

VEGASYSVVILAGLGVAALAGYAVLKELIFEPKEYKIFGKALARVQSDSQVTAKIGYPITGYGTESRNRA

ARQRIQNRVWTDEDGVEHVEVAFHIRGPHGAGKVFAEMFKDNADRTWKFTFLLVEITSPRPAQIMLESYLPA

>C6TAF0 Mitochondrial import inner membrane translocase subunit Tim21 Glycine max

MFRRILSHHRTLSACAHNATTRSLATRRHAPNVPPPPSFLIPRSRPLSSKTSQSNEAAESSNKAKKDVANVEDPFSAPTYNIPEKPVTFVEGASYSVVILAGLGIAAAAGYAVFKELIFQPKEYKIYNKALKRIQDDGQVRVRIGFPITGYGQESRNRAARQRIPHRVWTDEEGVEHVEVNFYIRGPHGHGKVFAEMFKGTDNEWKFTYLIVEIRAPSTAQIILESYIPSYSPTK

>A0A0A0K9E8 Mitochondrial import inner membrane translocase subunit Tim21 Cucumis sativus

MANGLMKLTRFISPSSLLPRQWHHSSFSRLGSHEFLQTAEVMRSSGMNASKRVTVNIIDEAKSGLPDTYATSVRELLAPLKSGISKQSAVYVCDIPKAALGGMPVLSRWQDARASVVNFSTLGVTSRCDTSARGPCFARFMSSKSSEKRGQTESESKKEISTVEDPFDAPTYNIPEKPVTFAEGASYSFIILAGLGVAAAAGYAVFKELIFQPKEYKIFDKALKRIQDDSQVRVRIGSPITGYGQETRNRAARQRIPNRVWTDEDGVERVEVNFYIRGPHGAGKVYTEMFKDQVDKQWKFTYLIVEVKSPSPAQLILESYMPA

>A0A1U8IDF4 Gossypium hirsutum

MNRRSSSRSRWSGSRSVSKYDDASAGKRSAVGNSNTRYVTGANNMSKGVGATGRGHCGRDCVRASVHNHGSKSTCARSASRTSKSKTSTRKSNVDDATYNDKVTTGASYSVGGAAAAGYAVKKYKNARNSVRVRGHTGYGTRNRAARRNRYTDNGVHVVNYRGHGAGKVAMKDKTDNKWKYTYVNSSRAMSYAAMRSSTN

>A0A1U8KFX3 Mitochondrial import inner membrane translocase subunit Tim21 Gossypium hirsutum

MQNIRRSIISSRSRWSGSFRSFVESKLYLDDFALAAKRFSAVGNSNFTRQYVTGANNMSKGVLGATGIPLPFRGHFGRDWLVRFQASVPHIENHGSKVISTCFARSFASRTSKQSKETSETRKELSNVEDPFDAPTYNIPDKPVTFTEGASYSLIILVGLGIAAAAGYAVFKELIFQPKEYKIFNQALERIQNESQVRVRIGHPITGYGQETRNRAARQRIPNRIYTDENGVEHVEVNFYIRGPHGAGKVFAEMFKDKTDNKWKYTYLIVQINSPSRAELMLESYLPAAEMRSSTN
