# Supplementary material for: NLG1, encoding a mitochondrial membrane protein, controls leaf and grain development in rice
Source: BMC Plant Biol. 2023 Sep 9;23:418. doi: 10.1186/s12870-023-04417-2 (PMC10492415; doi:10.1186/s12870-023-04417-2)
Supplement: Supplementary file 4 — Supplementary Material 4 [file 12870_2023_4417_MOESM4_ESM.docx]

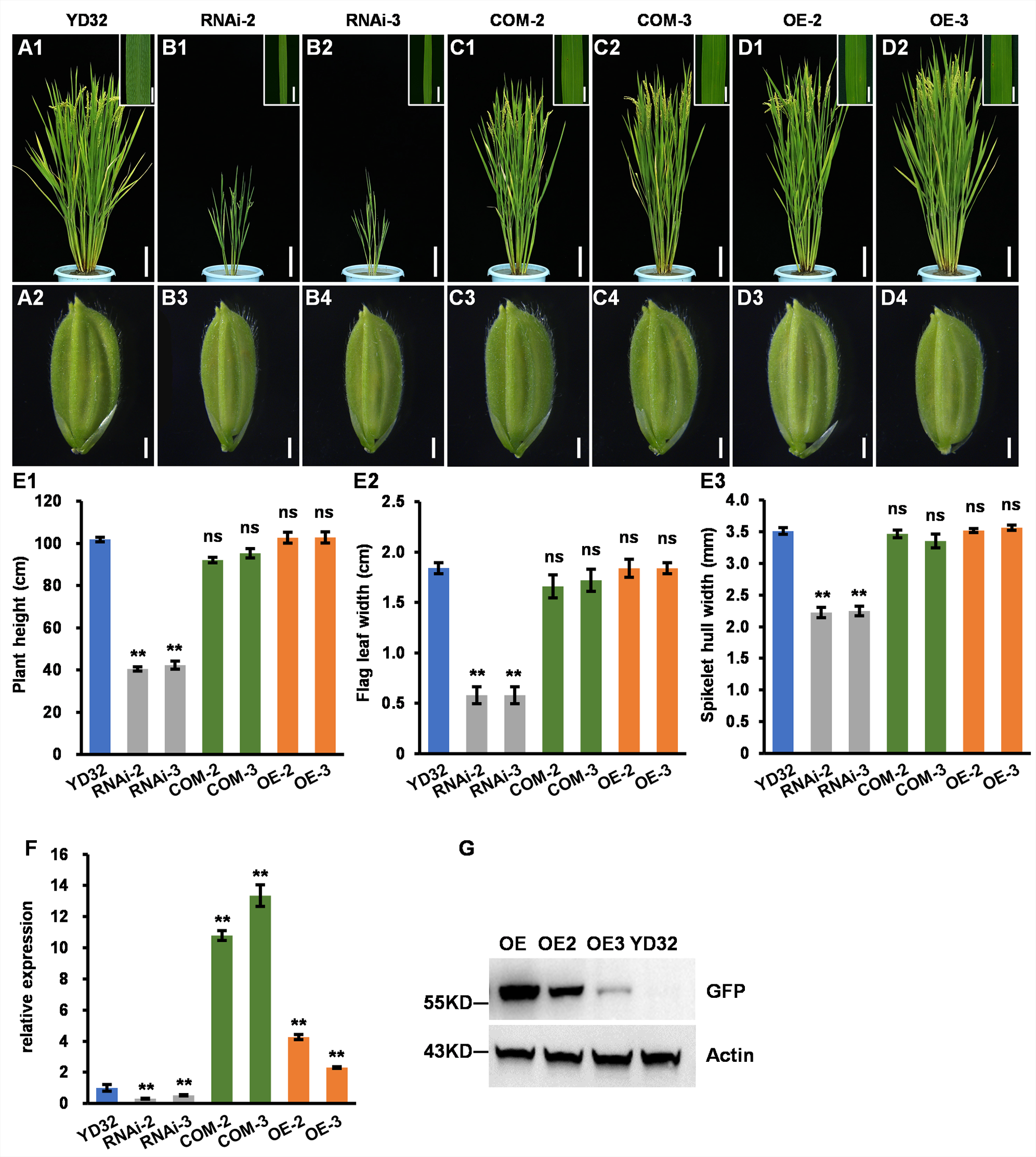


**Fig. S2.** Morphological comparison among YD32, RNAi, COM and OE transgenic lines at heading stage. **A-D** Morphological comparison of plant architecture, flag leaf and spikelet of YD32 (A1-A2), RNAi (*NLG1*-RNA interference) (B1-B4), COM (*NLG1*-complementation) (C1-C4), OE (*NLG1*-overexpression) (D1-D4). **E1-E2** Plant height (E1), flag leaf width (E2) and spikelet hull width (E3) of YD32, RNAi, COM and OE lines. Data represent means ± SD (*n* = 5). **F** Expression analysis of NLG1 in the flag leaves of the YD32, RNAi, COM and OE lines using RT-qPCR. Data represent means ± SD (*n* = 3). **G** Immunoblot showing the quantities of NLG1-GFP fusion protein in flag leaves from three independent OE lines. Actin showing approximately equal loading of total proteins. **Significant difference at p < 0.01 compared with YD32 by Student’s *t*-test, and ns means no significance. Scale bars: 10 cm in A1, B1, B2, C1, C2, D1, D2; 1 mm in flag leaf, 1 mm in A2, B3, B4, C3, C4, D3, D4.
