# Supplementary material for: NLG1, encoding a mitochondrial membrane protein, controls leaf and grain development in rice
Source: BMC Plant Biol. 2023 Sep 9;23:418. doi: 10.1186/s12870-023-04417-2 (PMC10492415; doi:10.1186/s12870-023-04417-2)
Supplement: Supplementary file 5 — Supplementary Material 5 [file 12870_2023_4417_MOESM5_ESM.docx]

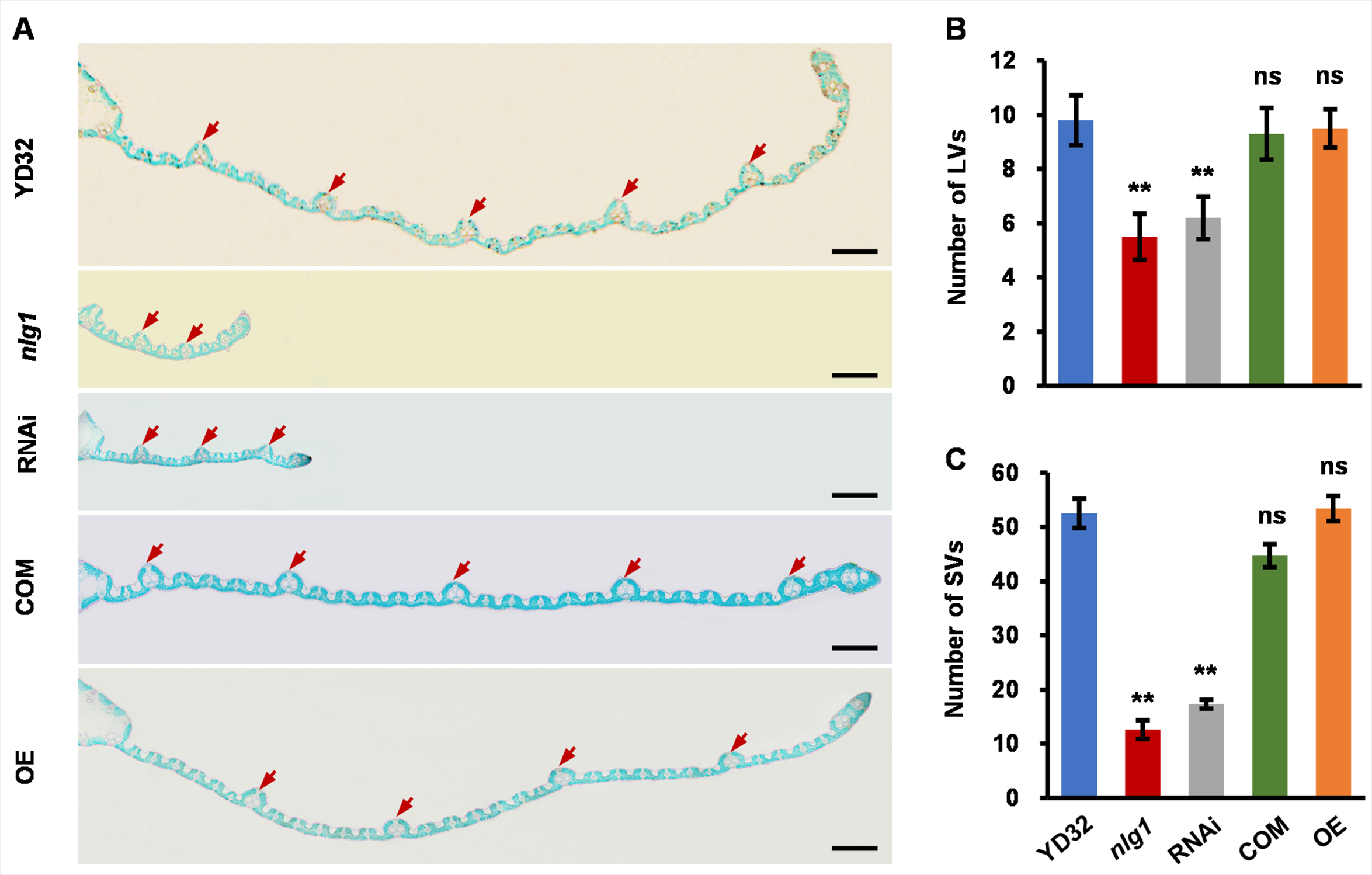


**Fig. S3.** Histological analysis of the flag leaves from YD32 and *nlg1*, RNAi, COM and OE transgenic lines at reproductive stage. **A** Paraffin transverse sections of the flag leaves of YD32, *nlg1*, RNAi, COM and OE. Red arrows point out the large vascular bundles (LVs). **B-C** Comparisons of the number of the large vascular bundles (LVs) B the small vascular bundles (SVs) C. Data represent means ± SD (*n* = 10). **Significant difference at p < 0.01 compared with YD32 by Student’s *t*-test, and ns means no significance. Scale bars: 500 μm in A.
