# Supplementary material for: NLG1, encoding a mitochondrial membrane protein, controls leaf and grain development in rice
Source: BMC Plant Biol. 2023 Sep 9;23:418. doi: 10.1186/s12870-023-04417-2 (PMC10492415; doi:10.1186/s12870-023-04417-2)
Supplement: Supplementary file 6 — Supplementary Material 6 [file 12870_2023_4417_MOESM6_ESM.docx]

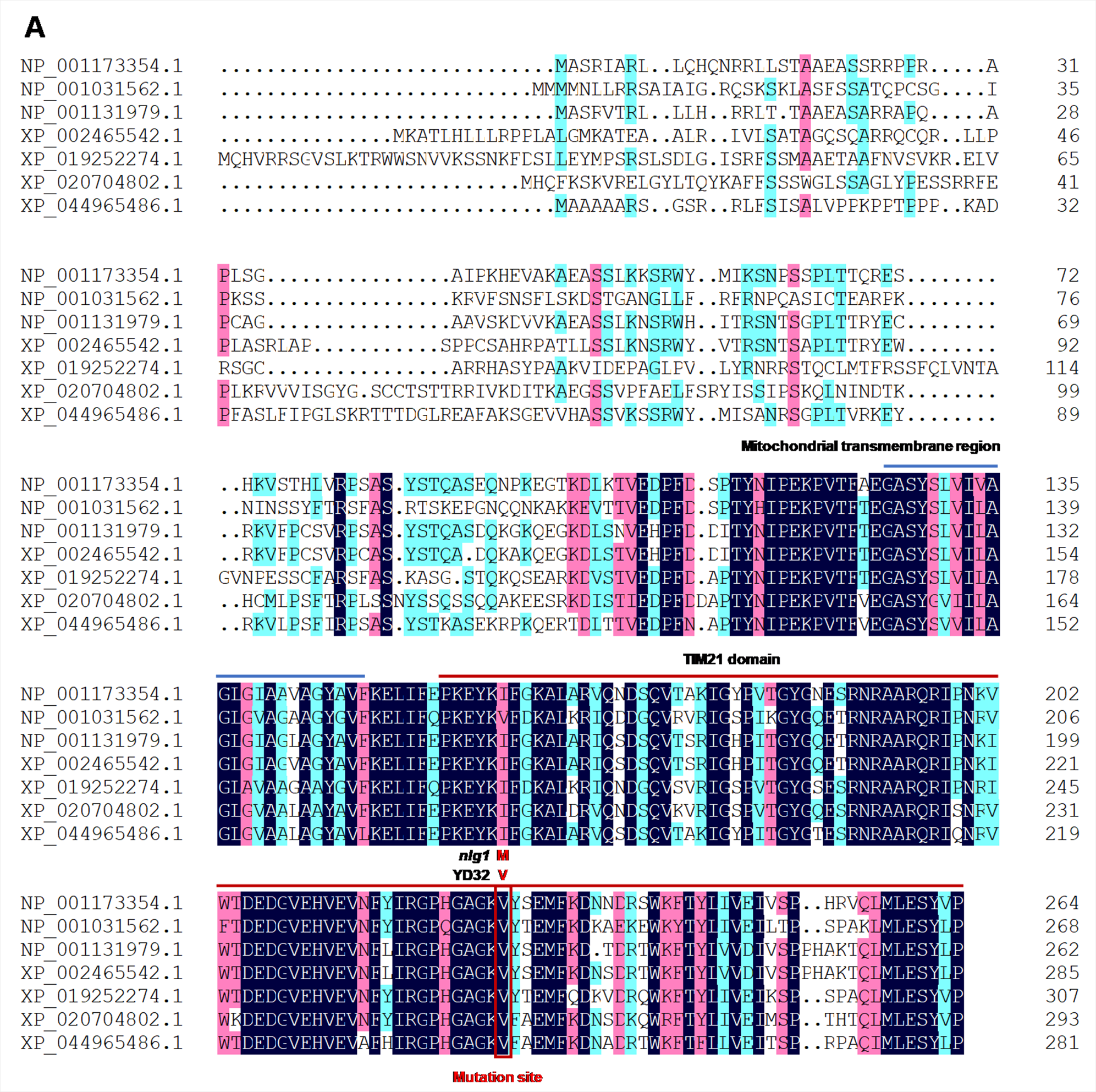


**Fig. S4.** Alignment of amino acid sequence NLG1. **A** Alignment of amino acid sequence of NLG1 with other 6 representative species in plants. Mitochondrial transmembrane region and TIM21 domain were indicated by blue line and red line, respectively. The mutation site was pointed out by red rectangle. *Oryza sativa Japonica Group* [GenBank accession number: NP_001173354.1, NLG1], *Arabidopsis thaliana* [GenBank accession number: NP_001031562.1], *Zea mays* [GenBank accession number: NP_001131979.1], *Sorghum bicolor* [GenBank accession number: XP_002465542.1], *Nicotiana attenuata* [GenBank accession number: XP_019252274.1], *Dendrobium catenatum* [GenBank accession number: XP_020704802.1], *Hordeum vulgare subsp. Vulgare* [GenBank accession number: XP_044965486.1].
