# Supplementary material for: NLG1, encoding a mitochondrial membrane protein, controls leaf and grain development in rice
Source: BMC Plant Biol. 2023 Sep 9;23:418. doi: 10.1186/s12870-023-04417-2 (PMC10492415; doi:10.1186/s12870-023-04417-2)
Supplement: Supplementary file 7 — Supplementary Material 7 [file 12870_2023_4417_MOESM7_ESM.docx]

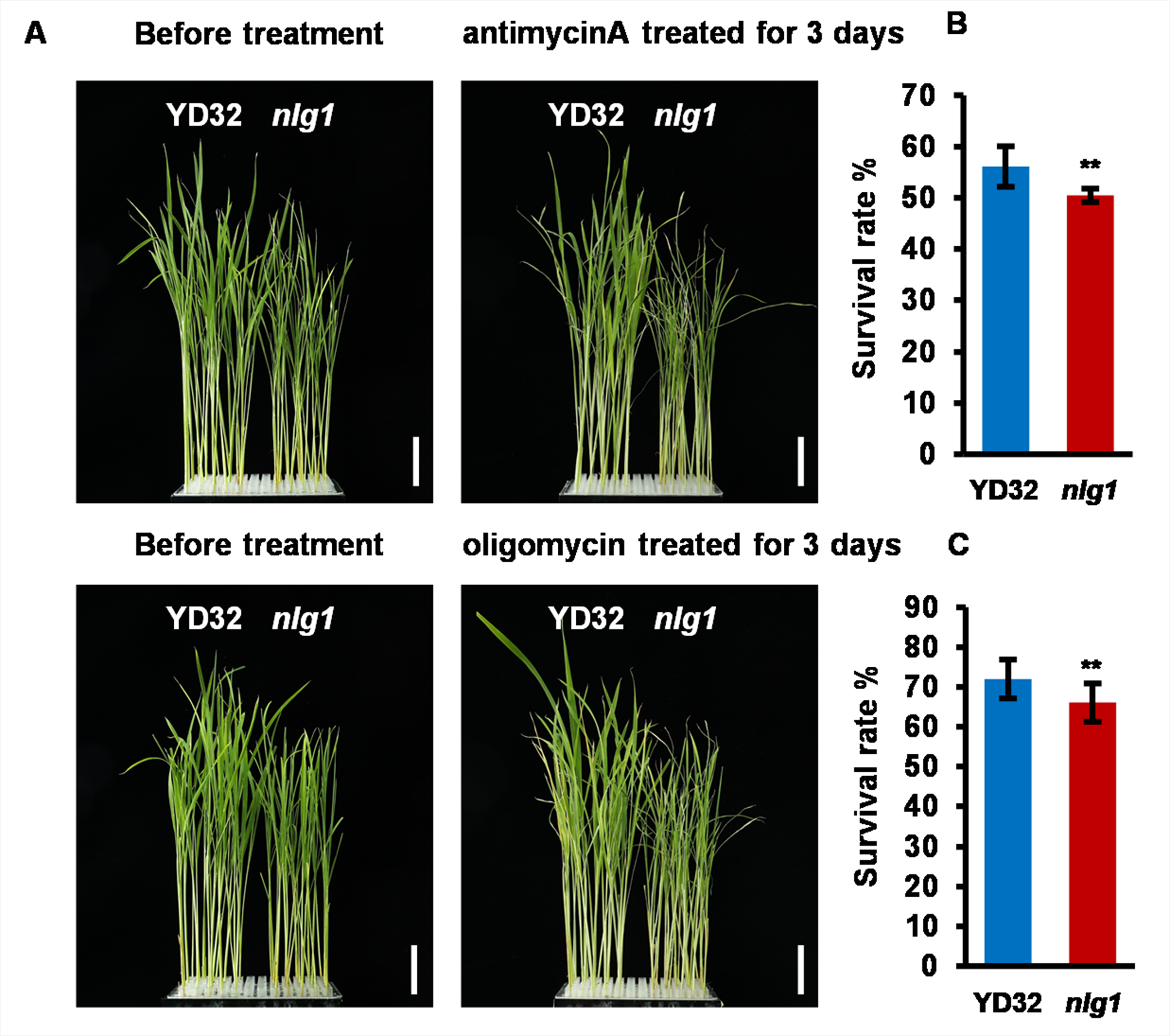


**Fig. S5.** Treatment with mitochondrial electron transport inhibitors. **A** Phenotypes of the YD32 and *nlg1* mutant after treatment with 1 μM antimycin A and 0.2 μM oligomycin for 3 days. **B-C** The survival rate of the YD32 and *nlg1* mutant after treatment with 1 μM antimycin A and 0.2 μM oligomycin, respectively. Data represent means ± SD (*n* = 6). **Significant difference at p < 0.01 compared with YD32 by Student’s *t*-test. Scale bars: 5 cm in A.
