# Supplementary material for: NLG1, encoding a mitochondrial membrane protein, controls leaf and grain development in rice
Source: BMC Plant Biol. 2023 Sep 9;23:418. doi: 10.1186/s12870-023-04417-2 (PMC10492415; doi:10.1186/s12870-023-04417-2)
Supplement: Supplementary file 8 — Supplementary Material 8 [file 12870_2023_4417_MOESM8_ESM.docx]

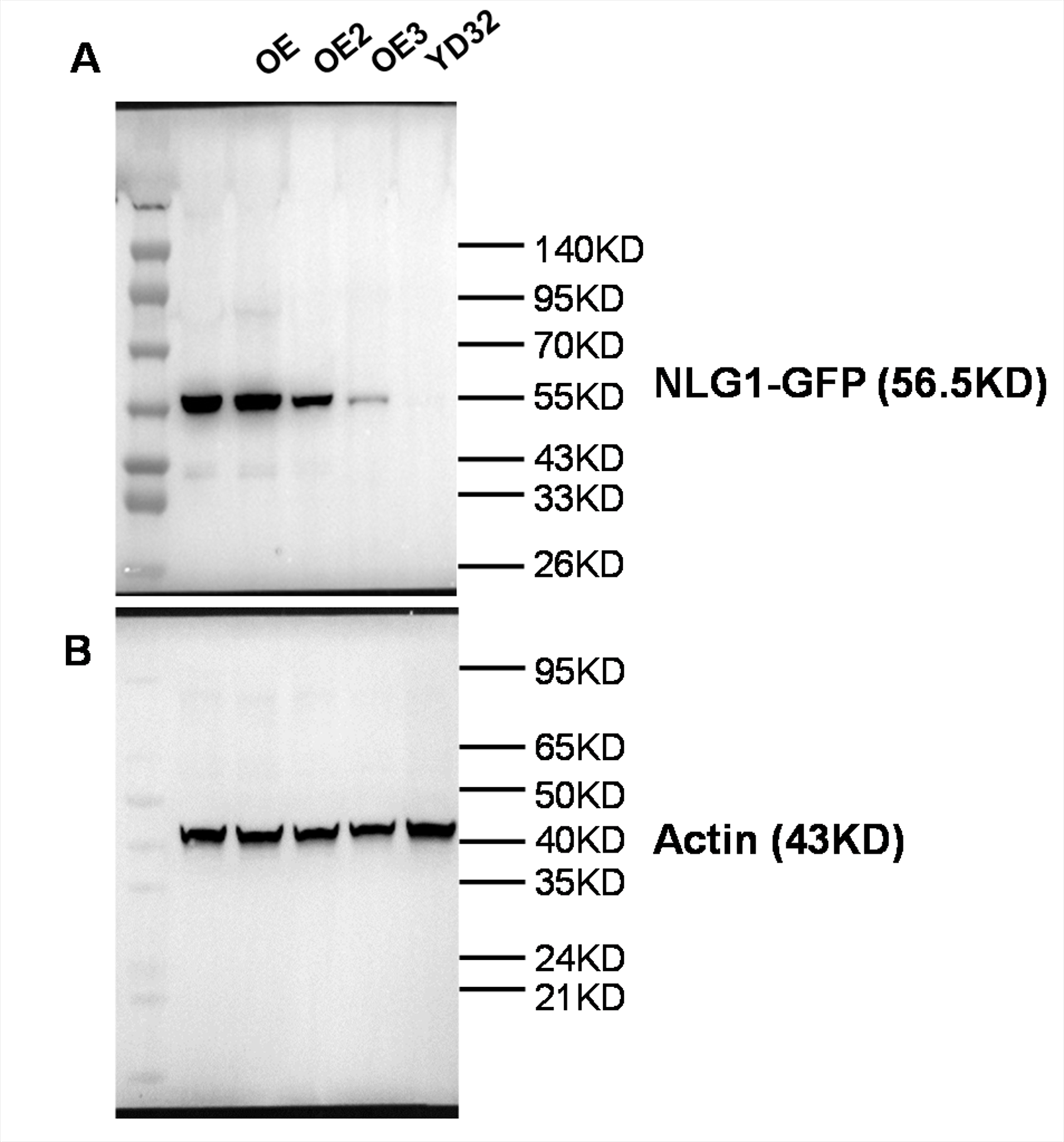


**Supporting Fig. 1.** Original images of blots. **A** Detection of NLG1 expression in OE (*NLG1*-overexpression) transgenic lines at heading stage. **B** Actin acted as the internal reference.
