# Supplementary material for: NLG1, encoding a mitochondrial membrane protein, controls leaf and grain development in rice
Source: BMC Plant Biol. 2023 Sep 9;23:418. doi: 10.1186/s12870-023-04417-2 (PMC10492415; doi:10.1186/s12870-023-04417-2)
Supplement: Supplementary file 9 — Supplementary Material 9 [file 12870_2023_4417_MOESM9_ESM.docx]

***NLG1*, a novel mitochondrial membrane protein, control leaf and grain development in rice**

Yi Wen^1,2+^, Kaixiong Wu^2+^, Bingze Chai^2+^, Peng Hu^2^, Yiqing Tan^2^, Yueying Wang^2^, Hao Wu^2^, Junge Wang^2^, Li Zhu^2^, Guangheng Zhang^2^, Zhenyu Gao^2^, Deyong Ren^2^, Dali Zeng^2^, Lan Shen^2^, Guojun Dong^2^, Qiang Zhang^2^, Qing Li^2^, Qian Qian^1,2,3,4*^, Jiang Hu^2*^

^1^Rice Research Institute, Shenyang Agricultural University, Shenyang, China

^2^State Key Laboratory of Rice Biology, China National Rice Research Institute, Hangzhou, China

^3^Hainan Yazhou Bay Seed Laboratory, Sanya 572024, Hainan, China

^4^National Nanfan Research Institute (Sanya), Chinese Academy of Agricultural Sciences, Sanya 572024, China

^*^Corresponding author: qianqian188@hotmail.com (Q.Q); hujiang588@163.com (J.H)

^+^These authors have contributed equally to this work and share first authorship

**Supplementary Information**

**Fig. S1**. Dynamic characteristics of seedlings of YD32 and *nlg1*. **A-D** Representative images of YD32 (up) and *nlg1* (down) at different stage, 10 Days (A), 20 Days (B), 30 Days (C), 35 Days (D), Scale bars: 2 cm. E-F Leaf width (E) and plant height (F) of the YD32 and *nlg1* during 10 days to 35 days growth stage. Data represent means ± SD (*n* = 5).

**Fig. S2.** Morphological comparison among YD32*,* RNAi, COM and OE transgenic lines at heading stage. **A-D** Morphological comparison of plant architectures, flag leaves and spikelet hulls of YD32 (A1-A2), RNAi (*NLG1*-RNA interference) (B1-B4), COM (*NLG1*-complementation) (C1-C4), OE (*NLG1*-overexpression) (D1-D4). **E1-E2** Plant height (E1), flag leaf width (E2) and spikelet hull width (E3) of YD32, RNAi, COM and OE lines. Data represent means ± SD (*n* = 5). **F** Expression analysis of *NLG1* in the flag leaves of the YD32, RNAi, COM and OE lines using RT-qPCR. Data represent means ± SD (*n* = 3). **G** Immunoblot showing the quantities of NLG1-GFP fusion protein in flag leaves from three independent OE lines. Actin showing approximately equal loading of total proteins. **Significant difference at p < 0.01 compared with YD32 by Student’s *t*-test, and ns means no significance. Scale bars: 10 cm in A1, B1, B2, C1, C2, D1, D2; 1 mm in flag leaf, 1 mm in A2, B3, B4, C3, C4, D3, D4.

**Fig. S3.** Histological analysis of the flag leaves from YD32 and *nlg1,* RNAi, COM and OE transgenic lines. **A** Paraffin transverse sections of the flag leaves of YD32, *nlg1*, RNAi, COM and OE. Red arrows point out the large vascular bundles (LVs). **B-C** Comparisons of the number of the large vascular bundles (LVs) B, the small vascular bundles (SVs) C. Data represent means ± SD (*n* = 10). **Significant difference at p < 0.01 compared with YD32 by Student’s *t*-test, and ns means no significance. Scale bars: 500 μm in A.

**Fig. S4.** Alignment of NLG1 amino acid sequence. **A** Alignment of amino acid sequence of NLG1 with other 6 representative species in plants. Mitochondrial transmembrane region and TIM21 domain were indicated by blue line and red line, respectively. The mutation site was pointed out by red rectangle. *Oryza sativa Japonica Group* [GenBank accession number: NP_001173354.1, NLG1], *Arabidopsis thaliana* [GenBank accession number: NP_001031562.1], *Zea mays* [GenBank accession number: NP_001131979.1], *Sorghum bicolor* [GenBank accession number: XP_002465542.1], *Nicotiana attenuata* [GenBank accession number: XP_019252274.1], *Dendrobium catenatum* [GenBank accession number: XP_020704802.1], *Hordeum vulgare subsp. Vulgare* [GenBank accession number: XP_044965486.1].

**Fig. S5.** Treatment with mitochondrial electron transport inhibitors. **A** Phenotypes of the YD32 and *nlg1* mutant after treatment with 1-μM antimycin A and 0.2-μM oligomycin for 3 days. **B-C** The survival rate of the YD32 and *nlg1* mutant after treatment with 1-μM antimycin A and 0.2-μM oligomycin, respectively. Data represent means ± SD (*n* = 6). **Significant difference at p < 0.01 compared with YD32 by Student’s *t*-test. Scale bars: 5 cm in A.

**Table S1.** Annotation of the 6 ORFs in the 42.1-kb target region.

**Table S2.** Primers used in this study.

**Table S3.** Four groups of the DEGs between W7 and *nlg1* in the heatmap.


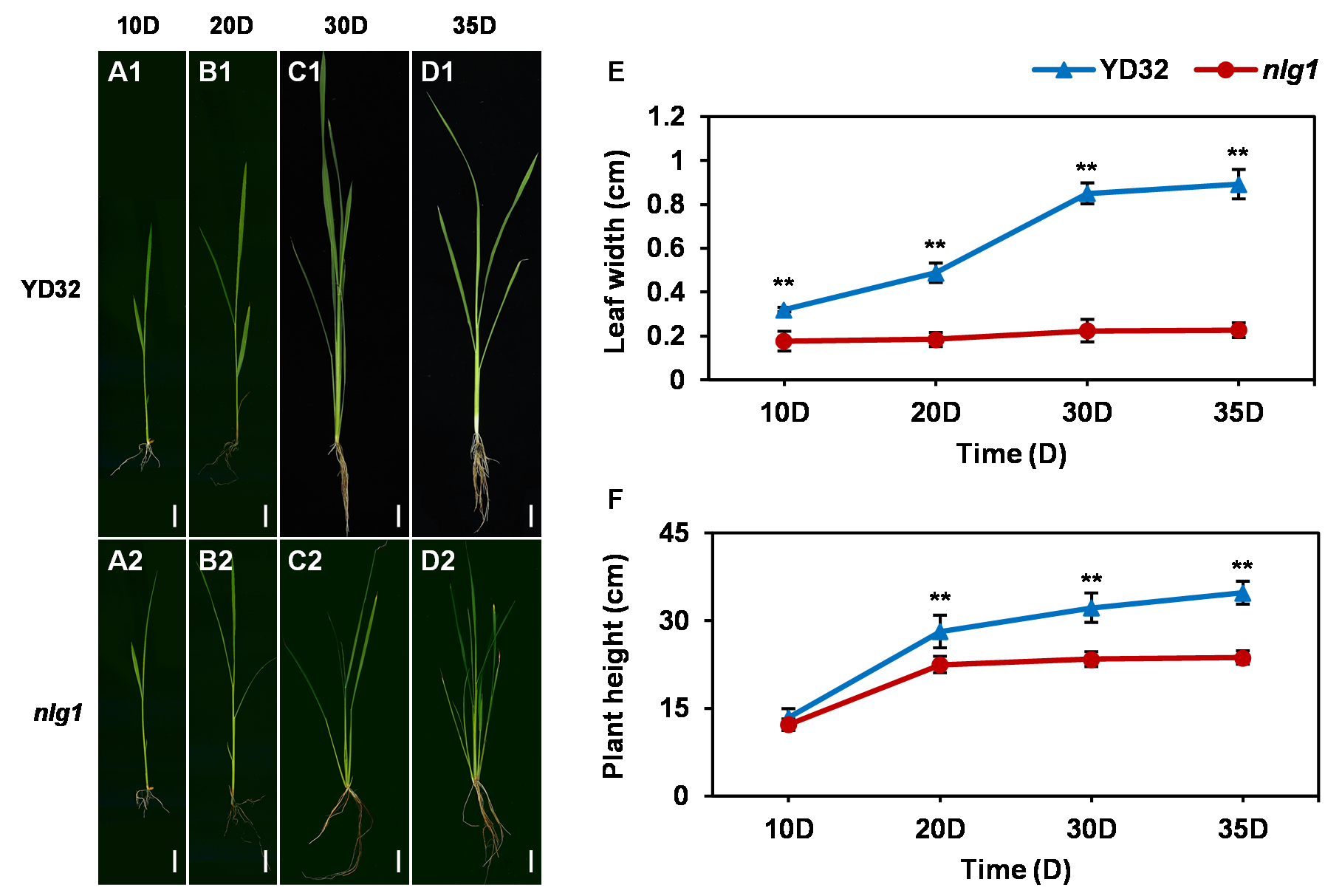


**Fig. S1.** Dynamic characteristics of seedling of YD32 and *nlg1*. **A-D** Representative images of YD32 (up) and *nlg1* (down) at different stage, 10 Days (A), 20 Days (B), 30 Days (C), 35 Days (D), Scale bars: 2 cm. **E-F** Leaf width (E) and plant height (F) of the YD32 and *nlg1* during 10 days to 35 days growth stage. Data represent means ± SD (*n* = 5).
