# Supplementary material for: NLG1, encoding a mitochondrial membrane protein, controls leaf and grain development in rice
Source: BMC Plant Biol. 2023 Sep 9;23:418. doi: 10.1186/s12870-023-04417-2 (PMC10492415; doi:10.1186/s12870-023-04417-2)
Supplement: Supplementary file 10 — Supplementary Material 10 [file 12870_2023_4417_MOESM10_ESM.docx]

Additional file 8

Table S3. Four groups of the DEGs between W7 and *nlg1* in the heatmap

|  | Gene_id | | Y32-1 | Y32-2 | Y32-3 | *nlg1*-1 | *nlg1*-2 | *nlg1*-3 | Gene_name | Gene_description |
| --- | --- | --- | --- | --- | --- | --- | --- | --- | --- | --- |
| Cellulose  synthase | | LOC_Os07g24190 | 25.15786 | 93.30176 | 37.20577 | 7.022122 | 7.816697 | 8.806457 | CELLULOSE SYNTHASE A3 | Similar to Cellulose synthase-7 |
|  |  | LOC_Os06g12460 | 7.216573 | 9.770945 | 7.446284 | 1.457849 | 1.695926 | 0.45392 | CELLULOSE SYNTHASE LIKE A3 | Similar to Cellulose synthase-like A1 |
|  |  | LOC_Os03g62090 | 13.26705 | 11.83494 | 8.518439 | 0.890679 | 0.681674 | 0.562854 | CELLULOSE SYNTHASE A5 | Similar to Cellulose synthase-6 |
|  |  | LOC_Os08g06380 | 6.521242 | 6.862029 | 4.870595 | 0.328796 | 0.231233 | 0.027562 | CELLULOSE SYNTHASE LIKE F6 | MLG (mixed-linkage glucan) synthase, Biosynthesis of MLG (cell wall polysaccharide |
|  |  | LOC_Os03g56060 | 2.539304 | 2.286735 | 1.036652 | 0 | 0.101391 | 0.114813 | CELLULOSE SYNTHASE LIKE C9 | CSLC9 |
| Auxin response and transport | | LOC_Os07g29310 | 2.235981 | 3.402021 | 1.036805 | 0 | 0 | 0 | SMALL AUXIN-UP RNA 30 | Auxin responsive SAUR protein family protein |
|  |  | LOC_Os02g49160 | 5.262646 | 5.027933 | 2.492385 | 0.677175 | 0.206419 | 0.140247 | IAA8 | Similar to Isoform 2 of Auxin-responsive protein IAA8 |
|  |  | LOC_Os09g38130 | 8.588545 | 10.46926 | 7.640349 | 1.560394 | 1.257817 | 1.220851 | PIN likes 7a | Auxin efflux carrier domain containing protein |
|  |  | LOC_Os01g09450 | 6.63593 | 17.92857 | 13.33296 | 3.475753 | 4.222571 | 2.463223 | IAA2 | Similar to Auxin-responsive protein IAA26 (Indoleacetic acid-induced protein 26) (Phytochrome-associated protein 1) |
|  |  | LOC_Os07g08460 | 18.85571 | 24.0964 | 14.62244 | 7.007156 | 4.327464 | 5.073522 | IAA24 | AUX/IAA protein family protein |
|  |  | LOC_Os06g22870 | 30.56488 | 31.60107 | 35.78438 | 19.16549 | 16.61441 | 16.91054 | IAA21 | AUX/IAA protein family protein |
|  |  | LOC_Os04g57610 | 16.0494 | 17.7275 | 16.37956 | 7.067191 | 7.254183 | 5.887379 | AUXIN RESPONSE FACTOR 8 | Transcription factor, Regulator for phosphate homeostasis |
|  |  | LOC_Os03g07920 | 7.230007 | 8.451214 | 6.054191 | 1.402878 | 2.400146 | 1.608538 | BIG GRAIN1 | Positive regulator of auxin response and transport, Regulation of grain size |
|  |  | LOC_Os12g41600 | 8.767009 | 7.627975 | 12.71383 | 0 | 0 | 0 | SMALL AUXIN-UP RNA 57 | Auxin responsive SAUR protein domain containing protein |
| Energy transport | | LOC_Os01g72230 | 1.921075 | 1.649341 | 1.744633 | 0 | 0.07313 | 0 | - | Arf GTPase activating protein family protein |
|  |  | LOC_Os11g20790 | 8.78006 | 5.002223 | 5.432225 | 0.481827 | 0.665821 | 0.439811 | ADK-B | Adenylate kinase B (EC 2.7.4.3) (ATP-AMP transphosphorylase) |
|  |  | LOC_Os04g02670 | 102.7688 | 75.03534 | 131.7246 | 22.12178 | 29.7597 | 27.37412 | mitochondrial proton-transporting ATP synthase 1 | ATPase, F0 complex, subunit E, mitochondrial domain containing protein |
|  |  | LOC_Os08g15170 | 40.52549 | 30.20481 | 41.2787 | 18.38787 | 11.10844 | 13.61297 | - | ATPase, F1 complex, epsilon subunit, mitochondrial family protein |
|  |  | LOC_Os02g48720 | 123.703 | 105.6228 | 141.996 | 65.85217 | 64.16712 | 68.40957 | ADENOSINE NUCLEOTIDE TRANSLOCATOR | ADP, ATP carrier protein, mitochondrial precursor (ADP/ATP translocase) (Adenine nucleotide translocator) (ANT) |
| Mitochondrial membrane | | LOC_Os02g03880 | 5.712885 | 5.409392 | 5.095931 | 19.89543 | 18.80199 | 19.06461 | - | Similar to Mitochondrial import receptor subunit TOM22 homolog (Translocase of outer membrane 22 kDa subunit homolog) (TOM9) |
|  |  | LOC_Os03g19290 | 1.452072 | 1.30991 | 2.110852 | 4.0828 | 2.04746 | 2.627634 | - | Mitochondrial import inner membrane translocase, subunit Tim17/22 family protein |
|  |  | LOC_Os11g34760 | 1.966608 | 1.838797 | 1.77241 | 2.634413 | 2.540703 | 4.508792 | - | Mitochondrial matrix Mmp37 domain containing protein |
|  |  | LOC_Os01g62570 | 0.041745 | 0.070802 | 0.42796 | 3.241062 | 2.448645 | 3.199378 | - | Similar to Mitochondrial Rho GTPase，mitochondrial outer membrane protein |
|  |  | LOC_Os02g45100 | 0 | 0.031194 | 0.18855 | 1.147695 | 1.355445 | 1.691497 | - | Similar to Mitochondrial import inner membrane translocase subunit tim23 |
|  |  | LOC_Os03g30200 | 0.073074 | 0 | 0.107019 | 0.848361 | 1.428768 | 1.057862 | - | Mitochondrial import inner membrane translocase, subunit Tim17/22 family protein |
